# Sandwich Variance Estimation in Scale Mixture of Skew‐Normal Linear Mixed Models

**DOI:** 10.1002/sim.70753

**Published:** 2026-10-09

**Authors:** Keyliane Travassos, Larissa A. Matos, Fernanda L. Schumacher

**Affiliations:** ^1^ Departamento de Estatística Universidade Estadual de Campinas (UNICAMP) Campinas São Paulo Brazil; ^2^ Division of Biostatistics, College of Public Health The Ohio State University Columbus Ohio USA

**Keywords:** linear mixed‐effects model, sandwich variance estimator, scale mixture of skew‐normal distributions

## Abstract

Linear mixed models are often used to analyze studies with repeated measurements over time, where the data present a correlation between observations of the same individual. However, one of the main challenges in using these models is correctly defining the covariance structure associated with error and random effects. To overcome this challenge, we obtain the sandwich variance estimator as a robust alternative to estimate the standard error in the scale mixture of skew‐normal linear mixed models (SMSN‐LMMs). By definition, SMSN‐LMMs can accommodate skewness and heavy tails, and by considering our proposed variance estimator, we increase the model's flexibility by allowing for misspecification of the covariance components. We present the definition of the estimator, its derivation for this model, and its practical implementation. In addition, through simulation studies, we evaluate its performance in different scenarios, demonstrating its ability to provide reliable inferences even when the model or the covariance structure is misspecified.

## 1 Introduction

Linear mixed models are widely used to analyze data that exhibit some dependence between observations, such as longitudinal data. These models incorporate fixed effects that model the expected value of the variable of interest and random effects that capture the dependence and variability within each sampling unit. However, in practice, correctly specifying the covariance matrix of errors and random effects can be difficult. For a more detailed discussion of linear mixed models, see, for example, [1] and [2].

Several linear mixed models have been proposed in the literature, such as hierarchical models, used to analyze grouped data, such as students within classes or patients within hospitals [3, 4]; generalized linear mixed models that provide a more flexible approach to analyzing data that are not normally distributed, such as binary and count data, through the use of appropriate link functions [5, 6]; and linear mixed models that consider asymmetric distributions for errors and/or random effects have received attention, presenting greater flexibility in modeling data with asymmetry, heterogeneity or the presence of outliers (see, for example, [7, 8, 9]).

One of the difficulties encountered when using linear mixed models is estimating the standard errors of the model parameters. Violating classical assumptions such as the distribution of random effects and errors, or misspecification of the covariance structure, can lead to inappropriate statistical inferences and biased estimates of the standard error (see [10, 11]). To address this problem, several authors have proposed alternatives to obtain an estimate of the standard error that is robust to the misspecification of the variance and correlation functions. In this context, the term *robust* refers to the fact that standard errors are not greatly affected by violations of the correct specification of the covariance function.

Eicker [12] presented asymptotic results for models with heteroscedastic and dependent errors, providing a robust estimate of the variance. Huber [13] expanded on these ideas and proposed robust estimation methods for dealing with data that violate classical assumptions such as homoscedasticity or independence between observations. White [14] formalized what became known as the sandwich variance estimator, or robust variance estimator, which provides a consistent estimate of standard errors even when the covariance structure is misspecified. White [15] extended and popularized the use of this estimator in the context of linear regression with heteroscedastic errors. In addition, resampling‐based methods, such as bootstrap, are also used to obtain robust estimates of variance; see, for example, [16, 17], and [18]. Other proposals to improve inference in mixed linear models can be found in Harville, Liang and Zeger [19, 20], and Kenward and Roger [21].

The sandwich estimator is especially useful in the analysis of longitudinal data, where observations are collected repeatedly over time in each experimental unit, and it is common for there to be some degree of correlation between the errors of the observations of each individual. Its main advantages include robustness against misspecification of the error structure and its consistency in the presence of heteroscedasticity [14]. However, it can present greater variability, especially in small samples, compromising statistical inference [22], and it does not correct possible biases in the regression coefficients when the model is misspecified [23].

This work aims to develop a sandwich variance estimator for the flexible scale mixture of skew‐normal linear mixed models (SMSN‐LMM, [9]) based on the results presented by [15]. The SMSN class of distributions covers distributions such as skew‐normal (SN), skew‐t (ST), skew‐slash (SSL), and skew‐contaminated normal (SCN) and their symmetric versions. We investigated the performance of this estimator in different scenarios, seeking to understand in which situations the method provides reliable estimates and to identify possible limitations.

The rest of this manuscript is organized as follows. Section 2 presents the definition of SMSN‐LMM, as well as the formulation and derivation of the sandwich variance estimator for this model. Section 3 discusses the simulation studies conducted to evaluate the performance of the proposed estimator. Furthermore, comparisons are made with other interval estimation methods, including Monte Carlo methods, asymptotic methods, and bootstrap. Finally, Section 4 presents an illustration using a real dataset to exemplify the usefulness of the proposed estimator.

## 2 Methods

### 2.1 Background and Notation

Let $Y_{i}$ be a vector $n_{i}\times 1$ of continuous responses observed in the ith individual. The general linear mixed model is defined as

(1) $$Y_{i}=X_{i}\beta +Z_{i}b_{i}+\epsilon_{i},\;\;i=1,\ldots ,n,$$

where β is a l×1 vector of the population‐average regression coefficient, called fixed effects, $X_{i}$ is a $n_{i}\times l$ design matrix corresponding to the fixed effects, $Z_{i}$ is the $n_{i}\times q$ design matrix for the q×1 vector of random effects $b_{i}$, and $\epsilon_{i}$ is a $n_{i}\times 1$ vector of random errors.

Following Schumacher et al. Schumacher et al. [9], the SMSN‐LMM is formulated considering that

(2) $$\left(\begin{array}{l} b_{i}\\ \epsilon_{i} \end{array}\right)\overset{ind.}{∼}{\text{SMSN}}_{q+n_{i}}\left(\left(\begin{array}{l} c\Delta \\ 0 \end{array}\right),\left(\begin{array}{ll} D & 0\\ 0 & \Sigma_{i} \end{array}\right),\left(\begin{array}{l} \lambda \\ 0 \end{array}\right);H\right),\;\;i=1,\ldots ,n,$$

where $c=c(\nu )=-\sqrt{\frac{2}{\pi }}k_{1}$, with $k_{1}=E\{U^{-1/2}\}$ for a random variable U that assumes positive values, such that P(U≤u)=H(u;ν), D=D(α) is a q×q covariance matrix that depends on unknown and reduced parameter vector α, $\Delta =D^{1/2}\delta$, with $\delta =\lambda /\sqrt{1+\lambda^{⊤}\lambda }$; and $\Sigma_{i}=\sigma_{e}^{2}R_{i}$ is a covariance matrix of dimension $n_{i}\times n_{i}$, with $R_{i}(\phi )$ being a structured matrix depending on $\phi ={\left(\phi_{1},\ldots ,\phi_{p}\right)}^{⊤}$. Table 1 of Schumacher et al. [9] presents the expressions for $k_{1}$ obtained according to the distribution considered.

The proposed framework considers three possible dependence structures for modeling the error scale matrix: (i) *Uncorrelated* (UNC), with $R_{i}=I_{n_{i}}$; (ii) *Autoregressive dependence of order* p(AR(p), [24]), with $\phi =(\phi_{1},\ldots ,\phi_{p})$; and (iii) *Damped exponential correlation* (DEC, [25]), with $R_{i}=R_{i}(\phi ,t_{i})=[\phi_{1}^{|t_{ij}-t_{ik}{|}^{\phi_{2}}}],\;0\leq \phi_{1}<1,\;0<\phi_{2}<1,$ where $j,k=1,\ldots ,n_{i}$, for i=1,…,n, and $\phi ={\left(\phi_{1},\phi_{2}\right)}^{⊤}$.

As shown by Schumacher et al. Schumacher et al. [9], under the model specified in (1) and (2), $E(Y_{i})=X_{i}\beta$, with the marginal distribution of $Y_{i}$ being as follows:

$$Y_{i}\overset{ind.}{∼}{\text{SMSN}}_{n_{i}}({\text{X}}_{i}\beta +{\text{Z}}_{i}c\Delta ,\Psi_{i},{\bar{\lambda }}_{i};H),$$

where $\Psi_{i}=\Sigma_{i}+{\text{Z}}_{i}\text{D}{\text{Z}}_{i}^{⊤}$, ${\bar{\lambda }}_{i}=\frac{\Psi_{i}^{-1/2}{\text{Z}}_{i}\text{D}\zeta }{\sqrt{1+\zeta^{⊤}\Lambda_{i}\zeta }}$, with $\zeta ={\text{D}}^{-1/2}\lambda$ and $\Lambda_{i}={\left({\text{D}}^{-1}+{\text{Z}}_{i}^{⊤}\Sigma_{i}^{-1}{\text{Z}}_{i}\right)}^{-1}$; and the corresponding marginal probability density function (pdf) is given by

$$\begin{array}{ll} f(y_{i}) & =2\int_{0}^{\infty }\phi_{n_{i}}(y_{i};{\text{X}}_{i}\beta +{\text{Z}}_{i}c\Delta ,u^{-1}\Psi_{i})\\ & \;\times \Phi \left(u^{1/2}{\bar{\lambda }}_{i}^{⊤}\Psi_{i}^{-1/2}\left(y_{i}-{\text{X}}_{i}\beta -{\text{Z}}_{i}c\Delta \right)\right)dH(u;\nu ). \end{array}$$

Then, given the observed sample $y={\left(y_{1}^{⊤},\ldots ,y_{n}^{⊤}\right)}^{⊤}$, we have that the log‐likelihood function for $\theta^{*}$ is written as

$$\ell (\theta^{*}|y)=\overset{n}{\underset{i=1}{\sum }}\ell_{i}(\theta^{*}|y)=\overset{n}{\underset{i=1}{\sum }}\log\left(f_{Y_{i}}(y_{i}|\theta^{*})\right),$$

where $\theta^{*}={\left(\beta^{⊤},\sigma_{e}^{2},\phi^{⊤},\alpha^{⊤},\lambda^{⊤},\nu^{⊤}\right)}^{⊤}$. To obtain maximum likelihood estimates for the SMSN‐LMM, the authors implemented an ECME algorithm. For a detailed discussion, see Schumacher et al. Schumacher et al. [9].

To facilitate obtaining the first and second derivatives of the log‐likelihood function in relation to $\theta =\theta^{*}\setminus \nu$, we will consider the reparameterization $D=F^{2}$, where F is the square root of D containing q(q+1)/2 distinct elements $\alpha_{b}={\left(\alpha_{1},\ldots ,\alpha_{q(q+1)/2}\right)}^{⊤}$. So, the log‐likelihood function of θ, given the observed sample $y={\left(y_{1}^{⊤},\ldots ,y_{n}^{⊤}\right)}^{⊤}$ and ν, is of the form $\ell (\theta )=\sum_{i=1}^{n}\ell_{i}(\theta ,\nu )$, where

$$\ell_{i}(\theta ,\nu )=\log2-\frac{n_{i}}{2}\log(2\pi )-\frac{1}{2}\log|\Psi_{i}|+\log K_{i},$$

with

$$K_{i}(\theta ,\nu )=\int_{0}^{\infty }u_{i}^{n_{i}/2}\exp\left\{-\frac{1}{2}u_{i}d_{i}\right\}\Phi \left(u_{i}^{1/2}A_{i}\right)dH(u_{i};\nu ),$$

where $d_{i}={\left(y_{i}-X_{i}\beta -cZ_{i}\Delta \right)}^{⊤}\Psi_{i}^{-1}(y_{i}-X_{i}\beta -cZ_{i}\Delta )$ and

(3) $$A_{i}=\frac{\lambda^{⊤}FZ_{i}^{⊤}\Psi_{i}^{-1}(y_{i}-X_{i}\beta -cZ_{i}\Delta )}{{\left(1+\lambda^{⊤}F^{-1}\Lambda_{i}F^{-1}\lambda \right)}^{1/2}}.$$

Therefore, we have that the score vector is given by

(4) $$\overset{n}{\underset{i=1}{\sum }}\frac{\partial \ell_{i}(\theta ,\nu )}{\partial \theta }=-\frac{1}{2}\overset{n}{\underset{i=1}{\sum }}\frac{\partial \log|\Psi_{i}|}{\partial \theta }+\overset{n}{\underset{i=1}{\sum }}\frac{1}{K_{i}}\frac{\partial K_{i}}{\partial \theta },$$

where $K_{i}=I_{i}^{\Phi }\left(\frac{n_{i}}{2}\right)$ and $\frac{\partial K_{i}}{\partial \theta }=I_{i}^{\phi }\left(\frac{n_{i}+1}{2}\right)\frac{\partial A_{i}}{\partial \theta }-\frac{1}{2}I_{i}^{\Phi }\left(\frac{n_{i}+2}{2}\right)\frac{\partial d_{i}}{\partial \theta }$, with

(5) $$\begin{array}{cc} I_{i}^{\phi }(w) & =\int_{0}^{\infty }u_{i}^{w}\exp\left\{-\frac{1}{2}u_{i}d_{i}\right\}\phi_{1}\left(u_{i}^{1/2}A_{i}\right)dH(u_{i};\nu ),\\ I_{i}^{\Phi }(w) & =\int_{0}^{\infty }u_{i}^{w}\exp\left\{-\frac{1}{2}u_{i}d_{i}\right\}\Phi \left(u_{i}^{1/2}A_{i}\right)dH(u_{i};\nu ), \end{array}$$

$\phi_{1}(\cdot )$ and Φ(·) denote the pdf and the distribution function of the standard univariate normal distribution, respectively. The derivatives of $\log|\Psi_{i}|$, $A_{i}$, and $d_{i}$, together with the results of the substitution of H for each distribution considered, are presented by Schumacher et al. [9].

The Hessian matrix is of the form

(6) $$\begin{array}{ll} \overset{n}{\underset{i=1}{\sum }}\frac{\partial^{2}\ell_{i}(\theta ,\nu )}{\partial \theta \partial \theta^{⊤}} & =-\frac{1}{2}\overset{n}{\underset{i=1}{\sum }}\frac{\partial^{2}\log|\Psi_{i}|}{\partial \theta \partial \theta^{⊤}}+\overset{n}{\underset{i=1}{\sum }}\frac{1}{K_{i}}\frac{\partial^{2}K_{i}}{\partial \theta \partial \theta^{⊤}}\\ & \;-\overset{n}{\underset{i=1}{\sum }}\frac{1}{K_{i}^{2}}\frac{\partial K_{i}}{\partial \theta^{⊤}}\frac{\partial K_{i}}{\partial \theta }, \end{array}$$

where

(7) $$\begin{array}{cc} \frac{\partial^{2}K_{i}}{\partial \theta \partial \theta^{⊤}} & =\;I_{i}^{\phi }\left(\frac{n_{i}+1}{2}\right)\frac{\partial^{2}A_{i}}{\partial \theta \partial \theta^{⊤}}-\frac{1}{2}I_{i}^{\phi }\left(\frac{n_{i}+3}{2}\right)\\ & \;\times \left(\frac{\partial d_{i}}{\partial \theta^{⊤}}+2A_{i}\frac{\partial A_{i}}{\partial \theta^{⊤}}\right)\frac{\partial A_{i}}{\partial \theta }\\ & \;-\frac{1}{2}I_{i}^{\Phi }\left(\frac{n_{i}+2}{2}\right)\frac{\partial^{2}d_{i}}{\partial \theta \partial \theta^{⊤}}-\frac{1}{2}I_{i}^{\phi }\left(\frac{n_{i}+3}{2}\right)\frac{\partial A_{i}}{\partial \theta^{⊤}}\\ & \;-\frac{1}{4}I_{i}^{\Phi }\left(\frac{n_{i}+4}{2}\right)\frac{\partial d_{i}}{\partial \theta^{⊤}}, \end{array}$$

with $I_{i}^{\phi }(w)$ and $I_{i}^{\Phi }(w)$ are as defined in (5). The second derivates of $\log|\Psi_{i}|$, $A_{i}$, and $d_{i}$ are presented in Appendix A.

### 2.2 Sandwich Variance Estimator

Following the approach of White [15], the sandwich variance matrix estimator (SVE) is defined as

$$C(\theta )=A{\left(\theta \right)}^{-1}B(\theta )A{\left(\theta \right)}^{-1},$$

evaluated in $\theta =\theta_{*}$, where

(8) $$\begin{array}{ll} A(\theta ) & =E\left\{\overset{n}{\underset{i=1}{\sum }}\frac{\partial^{2}\log f(y_{i},\theta )}{\partial \theta_{l}\partial \theta_{k}}\right\},\;\;\text{and}\;\\ B(\theta ) & =E\left\{\overset{n}{\underset{i=1}{\sum }}\frac{\partial \log f(y_{i},\theta )}{\partial \theta_{l}}\frac{\partial \log f(y_{i},\theta )}{\partial \theta_{k}}\right\}. \end{array}$$

The standard error of each parameter estimate is obtained by the square root of the diagonal elements of the matrix C(θ). The sandwich variance matrix is approximated by

(9) $$C_{n}(\theta )=A_{n}{\left(\theta \right)}^{-1}B_{n}(\theta )A_{n}{\left(\theta \right)}^{-1},$$

with

$$\begin{array}{ll} A_{n}(\theta ) & =\frac{1}{n}\overset{n}{\underset{i=1}{\sum }}\frac{\partial^{2}\log f(y_{i},\theta )}{\partial \theta_{l}\partial \theta_{k}},\;\text{and}\\ B_{n}(\theta ) & =\frac{1}{n}\overset{n}{\underset{i=1}{\sum }}\frac{\partial \log f(y_{i},\theta )}{\partial \theta_{l}}\frac{\partial \log f(y_{i},\theta )}{\partial \theta_{k}}. \end{array}$$

Under the regularity conditions presented in White [15], it can be shown that ${\hat{\theta }}_{n}$ follows an asymptotically normal distribution, that is,

(10) $$\sqrt{n}({\hat{\theta }}_{n}-\theta_{*})∼N(0,C(\theta_{*})),$$

where $\theta_{*}$ represents the true value of the parameter and $C(\theta_{*})$ is the asymptotic covariance matrix. Furthermore, it is possible to show that the sandwich variance estimator, denoted by $C_{n}({\hat{\theta }}_{n})$, converges almost surely to $C(\theta_{*})$ as the sample size tends to infinity, that is, 


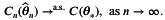


This estimator is robust against misspecification of the error structure, providing consistent estimates of the variance even when classical assumptions (such as homoscedasticity and independence of errors) are not satisfied. For more details and proof of the properties of this estimator, see White [14] and White [15].

The asymptotic results presented above depend on the regularity conditions presented in White [15], referred to by the author as the *“usual maximum likelihood regularity conditions*.” These assumptions include, for example, the identifiability of the parameter vector, the differentiability of the log‐likelihood function with respect to θ, the existence of finite first and second order moments of the score function, and the nonsingularity of the expected Hessian matrix A(θ). For SMSN‐LMMs, such conditions are expected to hold under the standard assumptions adopted for linear mixed models [1] and under the theoretical results established in Schumacher et al. [9]. However, a complete formal proof of these conditions for SMSN‐LMM is not trivial due to the complexity of the model structure. Therefore, we empirically assess the validity of the asymptotic results based on simulation studies.

To obtain the sandwich variance estimator in the context of the SMSN‐LMM, it is necessary to calculate the expectations indicated in (8), which are fundamental components in the definition of this estimator. These expectations, considering the SMSN‐LMM, can be expressed as follows:

(11) $$\begin{array}{cc} A(\theta ) & =-\frac{1}{2}E\left\{\overset{n}{\underset{i=1}{\sum }}\frac{\partial^{2}\log|\Psi_{i}|}{\partial \theta_{l}\partial \theta_{k}}\right\}+E\left\{\overset{n}{\underset{i=1}{\sum }}\frac{1}{K_{i}}\frac{\partial^{2}K_{i}}{\partial \theta_{l}\partial \theta_{k}}\right\}\\ & \;-E\left\{\overset{n}{\underset{i=1}{\sum }}\frac{1}{K_{i}^{2}}\frac{\partial K_{i}}{\partial \theta_{l}}\frac{\partial K_{i}}{\partial \theta_{k}}\right\};\\ B(\theta ) & =E\left\{\overset{n}{\underset{i=1}{\sum }}\frac{\partial \log|\Psi_{i}|}{\partial \theta_{l}}\frac{\partial \log|\Psi_{i}|}{\partial \theta_{k}}\right\}\\ & \;-E\left\{\overset{n}{\underset{i=1}{\sum }}\frac{\partial \log|\Psi_{i}|}{\partial \theta_{l}}\frac{1}{K_{i}}\frac{\partial K_{i}}{\partial \theta_{k}}\right\}+E\left\{\frac{1}{K_{i}^{2}}\overset{n}{\underset{i=1}{\sum }}\frac{\partial K_{i}}{\partial \theta_{l}}\frac{\partial K_{i}}{\partial \theta_{k}}\right\}. \end{array}$$

Since, the Hessian matrix and the product of the score for SMSN‐LMM involve complex expressions, calculating the expectations needed to determine the sandwich variance estimate can be complicated. Therefore, we will use the Monte Carlo method to facilitate this calculation. The scheme used to obtain a sandwich variance for the SMSN‐LMM is described in Algorithm 1.

ALGORITHM 1 **MC‐based estimation of sandwich variance for SMSN‐LMMs.**

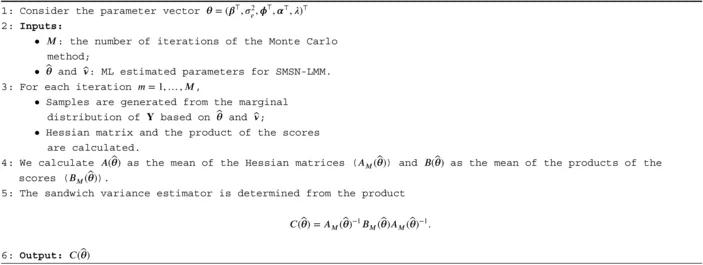

#### 2.2.1 Special Case: Scale Mixture of Normal

In the context of the scale mixture of normal linear mixed models (SMN‐LMM), we present in this section the results obtained with respect to fixed effects. Although, estimates for the expectations defined in (11) considering all model parameters can be obtained using Algorithm 1, restricting our focus to the fixed effects allows to simplify these expectations to the following form:

$$\begin{array}{ll} A(\beta ) & =E\left\{\overset{n}{\underset{i=1}{\sum }}\frac{1}{K_{i}}\frac{\partial^{2}K_{i}}{\partial \beta_{l}\partial \beta_{k}}\right\}-E\left\{\overset{n}{\underset{i=1}{\sum }}\frac{1}{K_{i}^{2}}\frac{\partial K_{i}}{\partial \beta_{l}}\frac{\partial K_{i}}{\partial \beta_{k}}\right\},\;\;\text{and}\;\;\\ B(\beta ) & =E\left\{\frac{1}{K_{i}^{2}}\overset{n}{\underset{i=1}{\sum }}\frac{\partial K_{i}}{\partial \beta_{l}}\frac{\partial K_{i}}{\partial \beta_{k}}\right\}. \end{array}$$

This restriction is particularly advantageous under the Normal and Student‐t distributions, as it admits closed‐form expressions for these expectations. In particular, for the case of the Student‐*t* distribution, the expectations are obtained by applying the result presented in Proposition 1. For the Slash and contaminated Normal distributions, where the calculation of expectations is more complex, we approximated using the Monte Carlo method, following the procedure described in Algorithm 1.

Proposition 1 *Let* $Y∼t_{p}(\mu ,\Sigma ,\nu )$ *. It can be shown, for* k=0,1, *and* 2*, that*:

$$E\left\{\frac{1}{{\left(\nu +d\right)}^{r}}Y^{\left(k\right)}\right\}=c_{p}(\nu ,r)E_{W}\left\{W^{\left(k\right)}\right\},\;\;W∼t_{p}(\mu ,\Sigma^{*},\nu +2r),$$

*where*

$$c(\nu ,r)={\left(\frac{1}{\nu }\right)}^{r}\left(\frac{\Gamma ((p+\nu )/2)\Gamma ((\nu +2r)/2)}{\Gamma (\nu /2)\Gamma ((p+\nu +2r)/2)}\right),$$

$d={\left(Y-\mu \right)}^{⊤}\Sigma^{-1}\left(Y-\mu \right)$, $\Sigma^{*}=(\nu /(\nu +2r))\Sigma$, $Y^{\left(0\right)}=1$, $Y^{\left(1\right)}=Y$, $Y^{\left(2\right)}=YY^{⊤}$ *, and* ν+2r>0.

Particular case of Proposition 2 presented by Matos et al. [26].

## 3 Simulation Studies

Simulation studies were conducted to evaluate the performance of the sandwich variance estimator and the impact of misspecification of the distribution of random effects or the correlation structure of random errors on this estimator. In addition, we evaluated differences in performance and estimate accuracy under different scenarios, and empirically evaluated the asymptotic results.

The sandwich variance estimator was obtained considering all the model parameters (SVE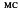) and considering only fixed effects (SVE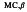). We also consider the asymptotic variance estimator (AVE) from the inverse of the observed information matrix, the bootstrap estimator (Bootstrap), and the Monte Carlo standard deviation (MC‐SD). In order to compare the results obtained with the scale mixture of normal linear mixed models, for which we obtained the exact expectations concerning fixed effects, we also calculate the inverse of the Fisher information matrix (IF), defined as $I(\theta )=-E\left\{\frac{\partial^{2}\log f(\text{y},\theta )}{\partial \theta \partial \theta^{⊤}}\right\}$.

Moreover, let ${\hat{\theta }}_{1},{\hat{\theta }}_{2},\ldots ,{\hat{\theta }}_{K}$ denote the estimates for a parameter θ obtained from K independent Monte Carlo replications of the simulation procedure. Its Monte Carlo standard deviation (MC‐SD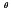) is defined as

$${\text{MC‐SD}}_{\theta }=\sqrt{\frac{1}{\left(K-1\right)}\overset{K}{\underset{k=1}{\sum }}{\left({\hat{\theta }}_{k}-\bar{\theta }\right)}^{2}},$$

where $\bar{\theta }=\frac{1}{K}\sum_{k=1}^{K}{\hat{\theta }}_{k}$ is the MC mean. This measure corresponds to the standard deviation of the replication estimates.

The simulations were performed using R software [27] on an Ubuntu 22.04.5 LTS system on a computer with an Intel Core i7 13700k processor with 16 GB of DDR4 RAM. To fit the models, we use the smn.lmm and smsn.lmm functions from the R package **skewlmm** [28], considering an EM‐type algorithm. In addition, we consider the confint function from the same package to obtain the confidence intervals using bootstrap and asymptotic methods. The results of the bootstrap and sandwich variance methods were obtained based on 100 simulated samples.

### 3.1 Simulation Study 1: Methods Comparison

To compare the results obtained by the AVE, bootstrap, and SVE with those generated by the MC‐SD, we generated 500 Monte Carlo samples from the following linear mixed model

(12) $$\begin{array}{ll} Y_{i} & =(\beta_{0}+b_{i})1_{6}+\beta_{1}x_{i1}+\beta_{2}x_{i1}^{2}+\beta_{3}\;x_{2i}\\ & \;+\beta_{4}\;x_{i1}\;x_{2i}+\epsilon_{i},\;\;i=1,\ldots ,n, \end{array}$$

where $x_{i1}={\left(-0.2,-0.1,0,0.1,0.2,0.3\right)}^{⊤}$, $x_{i1}^{2}={\left(x_{i1}^{2},\ldots ,x_{i6}^{2}\right)}^{⊤}$, and $x_{2i}$ is an all‐ones vector if the ith subject belongs to group 1, and an all‐zeros vector otherwise. For this study, we considered n=(20,50,100,300), and two parametrization scenarios for data generation:

a. $\beta_{0}=2.5$, $\beta_{1}=-1.5$, $\beta_{2}=6$, $\beta_{3}=-0.2$, $\beta_{4}=-0.15$, $b_{i}\overset{iid}{∼}\text{SN}(-0.5614,0.5,10)$ and $\epsilon_{i}\overset{ind}{∼}N_{6}(0,0.25R_{i})$, with $R_{i}$ being a 6×6 AR(1) dependence matrix with ϕ=0.6; and
b. $\beta_{0}=2.5$, $\beta_{1}=1.5$, $\beta_{2}=6$, $\beta_{3}=0$, $\beta_{4}=1$, $b_{i}\overset{iid}{∼}\text{SN}(-0.782,1,5)$ and $\epsilon_{i}\overset{ind}{∼}N_{6}(0,0.25R_{i})$, with $R_{i}$ being a 6×6 AR(1) dependence matrix with ϕ=0.3.

Figure 1 presents the mean interval lengths and coverage probabilities for the 95% confidence intervals under scenarios (a) and (b). The results are based on the Monte Carlo samples for parameters $\beta_{0}$, $\beta_{1}$, $\beta_{2}$, $\beta_{3}$, and $\beta_{4}$, obtained considering the methods AVE, bootstrap, and SVE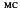, for different numbers of subjects. It can be seen that, in both scenarios, as n increases, the lengths of confidence intervals decreases for all methods. At the same time, the coverage probability, represented by the lines above the bars, generally remains close to 95% (indicated by the dashed line). In addition, for samples of size n=20, the asymptotic method tends to generate longer confidence intervals than the other methods. The results of the mean interval lengths and coverage probabilities for 95% confidence intervals obtained for all parameters are presented in Table A1 of Appendix B.

**FIGURE 1:**
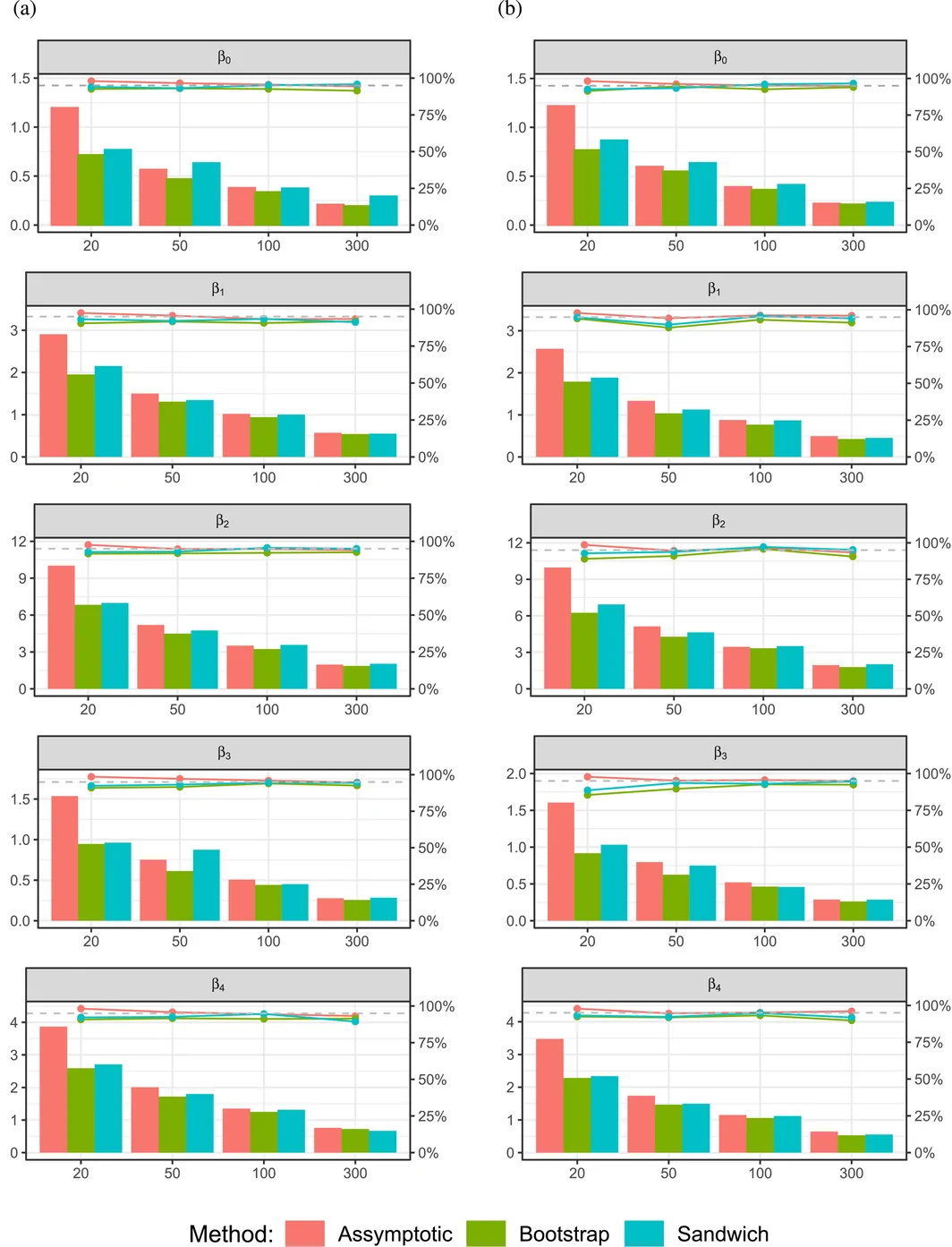
Simulation study 1. Mean interval lengths and coverage probabilities for 95% confidence intervals under scenarios (a) and (b). The results are based on 500 Monte Carlo samples, obtained considering asymptotic variance estimator (AVE), bootstrap, and sandwich variance estimator using Monte Carlo approximations (SVE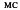), for different numbers of subjects (n). The dashed line represents the 95% coverage probability. (a) Scenario a. (b) Scenario b.

Table 1 shows the mean of the standard error estimates calculated for each parameter, obtained using the AVE, bootstrap, SVE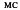, and SVE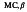 methods for different numbers of subjects. In addition, the table reports the MC‐SD of estimates for comparative purposes. The calculations were performed based on 500 Monte Carlo samples. We can observe that the methods considered present different behaviors according to the sample size. The asymptotic method tends to generate the largest standard errors, especially in small samples, whose estimates are further away from those obtained by the Monte Carlo method. On the other hand, the SVE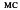 method presents a greater approximation to the Monte Carlo method in smaller samples, but still with some discrepancies, mainly for the λ parameter. Although, presenting greater variability for small samples, the bootstrap method approaches Monte Carlo estimates more as the sample size increases.

**TABLE 1:** Simulation study 1.

		Scenario (a)					Scenario (b)				
*n*	Parameter	MC‐SD	AVE	Bootstrap	SVE 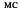	SVE 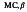	MC‐SD	AVE	Bootstrap	SVE 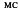	SVE 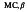
20	$\beta_{0}$	0.1989	0.3054	0.1825	0.2032	0.1936	0.2155	0.3109	0.1957	0.2218	0.1938
	$\beta_{1}$	0.5658	0.7411	0.4980	0.5087	0.5050	0.4730	0.6561	0.4566	0.4466	0.4462
	$\beta_{2}$	1.9106	2.5561	1.7440	1.8664	1.8546	1.8522	2.5436	1.5939	1.8483	1.8468
	$\beta_{3}$	0.2560	0.3920	0.2414	0.2561	0.2497	0.3004	0.4093	0.2341	0.2670	0.2565
	$\beta_{4}$	0.7267	0.9864	0.6941	0.6938	0.6813	0.6185	0.8862	0.5826	0.5933	0.5916
	$\sigma_{e}^{2}$	0.0328	0.0512	0.0306	0.0503	—	0.0364	0.0518	0.0301	0.0409	—
	ϕ	0.1269	0.2215	0.1225	0.2584	—	0.1356	0.2168	0.1445	0.1724	—
	F	0.2643	7.8426	0.2018	0.7757	—	0.2474	0.6708	0.2207	0.5025	—
	λ	6.4947	—	4.3066	19.2220	—	8.8418	—	6.6157	37.5958	—
50	$\beta_{0}$	0.1291	0.1443	0.1197	0.1407	0.1260	0.1362	0.1525	0.1401	0.1799	0.1275
	$\beta_{1}$	0.3471	0.3831	0.3337	0.3425	0.3386	0.3259	0.3403	0.2642	0.2946	0.2933
	$\beta_{2}$	1.2327	1.3255	1.1450	1.2206	1.2159	1.2629	1.3097	1.0933	1.2023	1.2004
	$\beta_{3}$	0.1675	0.1917	0.1563	0.1820	0.1652	0.1803	0.2031	0.1594	0.1988	0.1711
	$\beta_{4}$	0.4639	0.5123	0.4387	0.4574	0.4545	0.4146	0.4433	0.3742	0.3876	0.3859
	$\sigma_{e}^{2}$	0.0226	0.0266	0.0211	0.0639	—	0.0231	0.0260	0.0195	0.0396	—
	ϕ	0.0824	0.1041	0.0780	0.3632	—	0.0868	0.1021	0.0833	0.1879	—
	F	0.1997	0.7986	0.1581	2.3906	—	0.1718	0.2289	0.1471	0.6176	—
	λ	3.6687	—	2.5143	39.4235	—	5.0727	—	3.9204	149.8531	—
100	$\beta_{0}$	0.0906	0.0970	0.0860	0.0975	0.0908	0.0950	0.0996	0.0922	0.1066	0.0906
	$\beta_{1}$	0.2605	0.2600	0.2402	0.2461	0.2457	0.2147	0.2242	0.1955	0.2110	0.2110
	$\beta_{2}$	0.8795	0.8973	0.8252	0.8593	0.8593	0.8419	0.8803	0.8514	0.8479	0.8478
	$\beta_{3}$	0.1165	0.1292	0.1126	0.1199	0.1195	0.1230	0.1330	0.1180	0.1225	0.1224
	$\beta_{4}$	0.3505	0.3445	0.3188	0.3268	0.3268	0.2871	0.2937	0.2702	0.2748	0.2748
	$\sigma_{e}^{2}$	0.0163	0.0174	0.0153	0.0243	—	0.0160	0.0174	0.0166	0.0206	—
	ϕ	0.0577	0.0673	0.0557	0.1205	—	0.0553	0.0664	0.0631	0.0875	—
	F	0.1482	0.2673	0.1216	0.5252	—	0.1157	0.1355	0.1099	0.2531	—
	λ	2.7574	—	1.7722	13.6479	—	3.3148	—	3.1403	40.1428	—
300	$\beta_{0}$	0.0536	0.0535	0.0495	0.0605	0.0522	0.0541	0.0562	0.0540	0.0555	0.0522
	$\beta_{1}$	0.1496	0.1459	0.1384	0.1453	0.1442	0.1230	0.1259	0.1086	0.1236	0.1236
	$\beta_{2}$	0.5088	0.5015	0.4752	0.4972	0.4971	0.5092	0.4963	0.4564	0.4909	0.4909
	$\beta_{3}$	0.0698	0.0709	0.0653	0.0697	0.0689	0.0687	0.0734	0.0667	0.0709	0.0709
	$\beta_{4}$	0.2014	0.1939	0.1849	0.1930	0.1928	0.1571	0.1641	0.1350	0.1616	0.1616
	$\sigma_{e}^{2}$	0.0093	0.0097	0.0089	0.0257	—	0.0093	0.0097	0.0102	0.0095	—
	ϕ	0.0326	0.0369	0.0321	0.1528	—	0.0367	0.0363	0.0350	0.0359	—
	F	0.0867	0.1045	0.0723	0.5675	—	0.0674	0.0699	0.0644	0.0753	—
	λ	1.4530	—	0.9929	43.9620	—	1.8722	—	1.5527	3.7854	—

*Note:* Standard error estimated under scenarios (a) and (b). The results are based on 500 Monte Carlo samples, considering the MC‐SD and the mean of the standard error estimates under methods AVE, bootstrap, SVE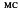, and SVE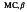 for different numbers of subjects (n). The data were generated using an AR(1)‐SN‐LMM.

Abbreviations: AVE, asymptotic variance estimator; MC‐SD, Monte Carlo standard deviation; SVE, sandwich variance estimator.

The differences between the methods decrease as the sample size increases. However, considerable variations are still observed, especially for the λ parameter, where the SVE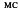 method presents the largest discrepancies. These results, which remain even as the sample size increases, serve as empirical evidence that the asymptotic convergence of the SVE method might not be attained. This could occur, for example, when estimates near the boundary compromise the score function's moments or the nonsingularity of the Hessian matrix A(θ), leading to numerical instabilities.

Table 2 presents the mean time in minutes to obtain the 95% confidence interval considering the methods AVE, bootstrap, SVE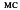, and SVE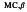 using different numbers of subjects based on the Monte Carlo samples. We can observe that for all considered methods, the execution time increases as the sample size increases, which is expected due to the greater computational complexity. The AVE method presented very low execution times (<0.0001 min), indicating that it is significantly faster than the other methods. It is also noted that when we use only fixed effects to obtain the SVE, there is a reduction in the time taken to obtain the estimator. Furthermore, it was observed that the bootstrap method has a significantly longer execution time than the other methods. Due to its higher computational cost, the bootstrap method will not be considered in the next simulations.

**TABLE 2:** Simulation study 1.

*n*	Method			
	AVE	Bootstrap	SVE 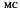	SVE 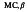
20	<0.0001	4.9751	0.3000	0.0881
50	<0.0001	8.9355	0.4550	0.1007
100	<0.0001	13.3276	0.7064	0.1213
300	<0.0001	30.7320	1.7899	0.2227

*Note:* Mean time in minutes to obtain a 95% confidence interval based on 500 Monte Carlo samples considering the methods AVE, bootstrap, and sandwich variance estimator, using all parameters (SVE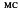) and using only fixed effects (SVE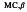), for different numbers of subjects (n). The data were generated using an AR(1)‐SN‐LMM.

Abbreviation: AVE, asymptotic variance estimator.

### 3.2 Simulation Study 2: Scale Mixture of Normal

To evaluate the performance of the sandwich variance estimator obtained for fixed effects considering exact expectations (SVE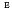), we compare its results with those obtained using Monte Carlo to approximate expectations. Furthermore, we also compare it with the results obtained by the AVE and IF methods. For this, we generate 500 Monte Carlo samples of the model presented in (1), considering $b_{i}\overset{iid}{∼}t_{1}(0,0.5,5)$ and $\epsilon_{i}\overset{ind}{∼}t_{6}(0,0.25R_{i},5)$, where $R_{i}$ is an 6×6 AR(1) dependence matrix with ϕ=0.6.

Table 3 presents the mean time in minutes to obtain a 95% confidence interval based on the Monte Carlo samples considering AVE, IF, SVE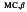, and SVE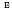 for different numbers of subjects. We can see that the AVE method presents low execution times (<0.0001 min), independent of the sample size. In contrast, the other methods show an increase in execution time as the sample size increases. However, it is noted that the SVE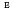 and IF methods have lower execution times than the SVE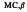 method, evidencing the gain, in terms of computational time, when using exact expectations.

**TABLE 3:** Simulation study 2.

*n*	Method			
	AVE	SVE 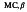	SVE 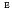	IF
20	<0.0001	0.1251	0.0003	0.0003
50	<0.0001	0.1312	0.0004	0.0004
100	<0.0001	0.1460	0.0007	0.0007
300	<0.0001	0.1991	0.0019	0.0019

*Note:* Mean time in minutes to obtain a 95% confidence interval based on 500 Monte Carlo samples considering the methods AVE, IF, and sandwich variance estimator for fixed effects, using Monte Carlo approximations (SVE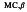) or exact expectations (SVE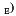, for different numbers of subjects (n). The data were generated using an AR(1)‐T‐LMM.

Abbreviations: AVE, asymptotic variance estimator; IF, inverse Fisher information.

Beyond that, Table 4 reports the mean of the standard error estimates based on the Monte Carlo samples obtained considering the methods AVE, IF, SVE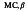, and SVE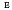 for different numbers of subjects. As a comparison, the table also reports the MC‐SD of the estimate. The results indicate that the estimates obtained by the SVE method are quite similar, both when using Monte Carlo approximations and when considering exact expectations. It is also observed that the values obtained by SVE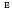 coincide exactly with those calculated by the inverse Fisher information. Furthermore, it is observed that for small samples, the AVE method tends to overestimate the standard error compared to the value obtained by Monte Carlo. As the number of subjects increases, the methods converge to values that are very close to each other.

**TABLE 4:** Simulation study 2.

		Method				
*n*	Parameter	MC‐SD	AVE	SVE 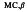	SVE 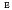	IF
20	$\beta_{0}$	0.3150	0.3577	0.2763	0.2777	0.2790
	$\beta_{1}$	0.6222	0.7742	0.5759	0.5819	0.5810
	$\beta_{2}$	2.1452	2.5376	2.0387	2.0192	2.0173
	$\beta_{3}$	0.4100	0.4972	0.3797	0.3791	0.3814
	$\beta_{4}$	0.8569	1.0263	0.7796	0.7671	0.7734
50	$\beta_{0}$	0.1797	0.1947	0.1814	0.1807	0.1817
	$\beta_{1}$	0.4082	0.4182	0.3818	0.3830	0.3836
	$\beta_{2}$	1.4097	1.4035	1.3119	1.3009	1.3115
	$\beta_{3}$	0.2558	0.2671	0.2472	0.2465	0.2479
	$\beta_{4}$	0.5385	0.5555	0.5152	0.5079	0.5106
100	$\beta_{0}$	0.1300	0.1353	0.1308	0.1301	0.1309
	$\beta_{1}$	0.2640	0.2835	0.2757	0.2740	0.2705
	$\beta_{2}$	0.9149	0.9642	0.9315	0.9308	0.9306
	$\beta_{3}$	0.1726	0.1844	0.1760	0.1762	0.1775
	$\beta_{4}$	0.3746	0.3770	0.3658	0.3624	0.3612
300	$\beta_{0}$	0.0750	0.0765	0.0752	0.0756	0.0761
	$\beta_{1}$	0.1618	0.1614	0.1595	0.1590	0.1589
	$\beta_{2}$	0.5814	0.5474	0.5423	0.5423	0.5419
	$\beta_{3}$	0.1094	0.1043	0.1027	0.1032	0.1033
	$\beta_{4}$	0.2205	0.2148	0.2127	0.2110	0.2113

*Note:* Standard error estimated. The results are based on 500 Monte Carlo samples, considering the MC‐SD and the mean of the standard error estimates under methods AVE, IF, and sandwich variance estimator for fixed effects, using Monte Carlo approximations (SVE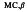) or exact expectations (SVE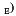, for different numbers of subjects (n). The data were generated using an AR(1)‐T‐LMM.

Abbreviations: AVE, asymptotic variance estimator; IF, inverse Fisher information; MC‐SD, Monte Carlo standard deviation.

### 3.3 Simulation Study 3: Investigating the Misspecification

In this section, we present a simulation study that investigates the behavior of the sandwich variance estimator under misspecification of the distribution of random effects and the correlation structure of random errors. Hence, we generated 500 Monte Carlo samples from the model

(13) $$\begin{array}{ll} Y_{i} & =(\beta_{0}+b_{0i})1_{10}+(\beta_{1}+b_{1i})x_{i1}+\beta_{2}\;x_{1i}^{2}+\beta_{3}\;x_{2i}\\ & \;+\beta_{4}\;x_{i1}\;x_{2i}+\epsilon_{i},\;\;i=1,\ldots ,n, \end{array}$$

where $\beta_{0}=2$, $\beta_{1}=-2$, $\beta_{2}=5$, $\beta_{3}=-0.04$, $\beta_{4}=0.06$, $x_{i}={\left(-0.2,-0.1,0.0,0.1,0.2,0.3\right)}^{⊤}$, $x_{i1}^{2}={\left(x_{i1}^{2},\ldots ,x_{i10}^{2}\right)}^{⊤}$, and $x_{2i}$ is an all‐ones vector if the ith subject belongs the group 1 and an all‐zeros vector otherwise. Let $R_{i}$ be an 6×6 AR(2) dependence matrix with $\phi_{1}=0.6$ and $\phi_{2}=0.2$ and $b_{i}={\left(b_{0i},b_{1i}\right)}^{⊤}$. For data generation, we consider n=(20,50,100,300),

(14) $$b_{i}\overset{iid.}{∼}t_{2}\left(\left(\begin{array}{c} 0\\ 0 \end{array}\right),\left(\begin{array}{cc} 1.5 & 1.3\\ 1.3 & 2.0 \end{array}\right),5\right),\;\text{and}\;\epsilon_{i}\overset{ind.}{∼}t_{6}(0,0.25\;R_{i},5).$$

Taking into account the results presented previously, which indicate greater computational efficiency when obtaining sandwich variance only for fixed effects, and that for certain parameters the SVE did not achieve the expected performance, in addition considering the interest in estimating standard errors of the fixed effects to carry out hypothesis tests and construct confidence intervals, this study considers the use of SVE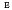 and SVE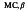. Moreover, we consider the AVE and Monte Carlo methods for comparison.

#### 3.3.1 Misspecification of the Random Effects Distribution

In this section, we will evaluate the behavior of the sandwich variance estimator under misspecification of the random effects distribution. The data were generated using an AR(2)‐T‐LMM and fitted using the T‐LMM, N‐LMM, and ST‐LMM models, all with AR(2) structure. Table 5 reports the mean of the standard error estimates in each of these models based on 500 Monte Carlo samples, considering the methods AVE and SVE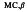, for different numbers of subjects. As in the previous simulations, the table also reports the MC‐SD of the estimates for comparison.

**TABLE 5:** Simulation study 3.

		Method						
			AVE			SVE 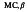		
*n*	Parameter	MC‐SD	T	N	ST	T	N	ST
20	$\beta_{0}$	0.4759	0.6524	0.9876	0.8791	0.4422	0.5241	0.3888
	$\beta_{1}$	0.8029	1.0982	1.6906	1.5132	0.7336	0.8627	0.6930
	$\beta_{2}$	2.0469	2.5752	3.7726	2.7862	1.7412	2.0452	1.6964
	$\beta_{3}$	0.6847	0.9467	1.4286	1.1209	0.6279	0.7263	0.5520
	$\beta_{4}$	1.1151	1.5138	2.3149	1.8356	1.0079	1.1926	0.9493
50	$\beta_{0}$	0.3260	0.3303	0.4666	0.3719	0.2999	0.3580	0.2911
	$\beta_{1}$	0.4819	0.5427	0.7574	0.6131	0.4872	0.5853	0.4775
	$\beta_{2}$	1.2244	1.2594	1.6895	1.2744	1.1433	1.3427	1.1369
	$\beta_{3}$	0.4450	0.4666	0.6605	0.4771	0.4209	0.4958	0.4085
	$\beta_{4}$	0.6568	0.7483	1.0444	0.7729	0.6705	0.8022	0.6583
100	$\beta_{0}$	0.2097	0.2221	0.2896	0.2442	0.2123	0.2481	0.2099
	$\beta_{1}$	0.3342	0.3635	0.4709	0.3994	0.3490	0.4142	0.3460
	$\beta_{2}$	0.7770	0.8557	1.0981	0.8603	0.8181	0.9562	0.8167
	$\beta_{3}$	0.2861	0.3109	0.4076	0.3128	0.2949	0.3465	0.2919
	$\beta_{4}$	0.4617	0.5020	0.6523	0.5052	0.4809	0.5678	0.4769
300	$\beta_{0}$	0.1291	0.1249	0.1556	0.1366	0.1232	0.1454	0.1229
	$\beta_{1}$	0.2000	0.2058	0.2595	0.2235	0.2032	0.2432	0.2024
	$\beta_{2}$	0.4992	0.4851	0.6000	0.4870	0.4775	0.5655	0.4779
	$\beta_{3}$	0.1791	0.1750	0.2185	0.1752	0.1726	0.2040	0.1723
	$\beta_{4}$	0.2895	0.2829	0.3562	0.2823	0.2784	0.3320	0.2773

*Note:* Standard error estimated. The results are based on the Monte Carlo samples, considering the MC‐SD and the mean of the standard error estimates under methods AVE and SVE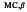, for different numbers of subjects (n). The data were generated using an AR(2)‐T‐LMM and fitted using the T‐LMM, N‐LMM, and ST‐LMM models, all with AR(2) structure.

Abbreviations: AVE, asymptotic variance estimator; MC‐SD, Monte Carlo standard deviation; SVE, sandwich variance estimator.

The results indicate that, in the presence of misspecification, the AVE method tends to overestimate the standard error compared to the value obtained by MC‐SD, with this overestimation being more pronounced when the adjustment is made with the N‐LMM model, followed by the ST‐LMM, especially in a smaller number of subjects. However, it is observed that as the number of subjects increases, this overestimation gradually decreases, and the performance of the AVE becomes more stable.

In contrast, the SVE method presents standard error estimates closer to the MC‐SD, even when the number of subjects is reduced, demonstrating greater robustness to misspecification. However, in some cases, a small overestimation is still observed, especially when the adjustment is performed using the N‐LMM model, which suggests that the method is not completely immune to the effects of misspecification of the distribution of random effects.

#### 3.3.2 Misspecified Covariance Matrix

To analyze the impact of the misspecification of the error covariance matrix on the estimation of sandwich variance, we estimate the generated data using an AR(2)‐T‐LMM, considering the UNC, AR(1), and AR(2) structures. Table 6 presents the results for this new scenario, using the same set of measures as in Simulation Section 3.3.1.

**TABLE 6:** Simulation study 3.

		Method						
			AVE			SVE 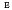		
*n*	Parameter	MC‐SD	AR(2)	AR(1)	UNC	AR(2)	AR(1)	UNC
20	$\beta_{0}$	0.4759	0.6524	0.6152	0.5810	0.4497	0.4498	0.4484
	$\beta_{1}$	0.8029	1.0982	1.0338	0.9626	0.7446	0.7442	0.7406
	$\beta_{2}$	2.0469	2.5752	2.4061	1.8141	1.7536	1.7545	1.5465
	$\beta_{3}$	0.6847	0.9467	0.8962	0.8513	0.6290	0.6304	0.6314
	$\beta_{4}$	1.1151	1.5138	1.4315	1.3589	1.0221	1.0215	1.0232
50	$\beta_{0}$	0.3260	0.3303	0.3256	0.3217	0.3000	0.2995	0.2988
	$\beta_{1}$	0.4819	0.5427	0.5329	0.5246	0.4875	0.4856	0.4857
	$\beta_{2}$	1.2244	1.2594	1.2279	0.9458	1.1525	1.1422	0.9887
	$\beta_{3}$	0.4450	0.4666	0.4612	0.4568	0.4193	0.4202	0.4209
	$\beta_{4}$	0.6568	0.7483	0.7350	0.7322	0.6695	0.6671	0.6723
100	$\beta_{0}$	0.2097	0.2221	0.2197	0.2179	0.2121	0.2114	0.2108
	$\beta_{1}$	0.3342	0.3635	0.3589	0.3561	0.3481	0.3464	0.3464
	$\beta_{2}$	0.7770	0.8557	0.8290	0.6351	0.8236	0.8103	0.6957
	$\beta_{3}$	0.2861	0.3109	0.3095	0.3080	0.2961	0.2967	0.2969
	$\beta_{4}$	0.4617	0.5020	0.4960	0.4974	0.4781	0.4761	0.4798
300	$\beta_{0}$	0.1291	0.1249	0.1238	0.1232	0.1237	0.1232	0.1229
	$\beta_{1}$	0.2000	0.2058	0.2035	0.2025	0.2024	0.2013	0.2013
	$\beta_{2}$	0.4992	0.4851	0.4637	0.3595	0.4788	0.4667	0.4025
	$\beta_{3}$	0.1791	0.1750	0.1750	0.1748	0.1726	0.1730	0.1732
	$\beta_{4}$	0.2895	0.2829	0.2802	0.2818	0.2780	0.2768	0.2789

*Note:* Standard error estimated. The results are based on 500 Monte Carlo samples, considering the MC‐SD and the mean of the standard error estimates under methods AVE and SVE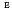, for different numbers of subjects (n). The data were generated using an AR(2)‐T‐LMM, and the T‐LMM was adjusted considering different dependence structures.

Abbreviations: AVE, asymptotic variance estimator; MC‐SD, Monte Carlo standard deviation; SVE, sandwich variance estimator.

We can observe that the AVE method tends to overestimate the standard error, especially in small samples. In contrast, SVE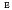 provides estimates closer to those obtained by MC‐SD, even when the dependence structure is misspecified or when the number of subjects is small, indicating the robustness of this estimator. Furthermore, we can highlight that misspecification has a greater impact in small samples and that the discrepancy between methods decreases as the number of subjects increases.

## 4 Illustration: Mice Weight Data

To exemplify the application of the sandwich variance estimator, we used the miceweight data set, which is included in the R package **skewlmm**. The data set was elaborated from a clinical trial to evaluate two diet treatments compared to a control group. The study was designed to evaluate whether any special diets promoted faster weight gain than the regular diet.

The experiment involved 52 mice, whose weight (in grams) was registered weekly over 10 weeks. After an initial period of four weeks without intervention, mice were randomly distributed into three groups: 21 mice started receiving the diet of treatment 1 (T1), 20 started receiving the diet of treatment 2 (T2), and the remaining 11 followed the standard diet in the control group (C). Figure 2 shows the weight profiles of the mice during the weeks of follow‐up for each diet considered. The mean profile observed in each group is highlighted.

**FIGURE 2:**
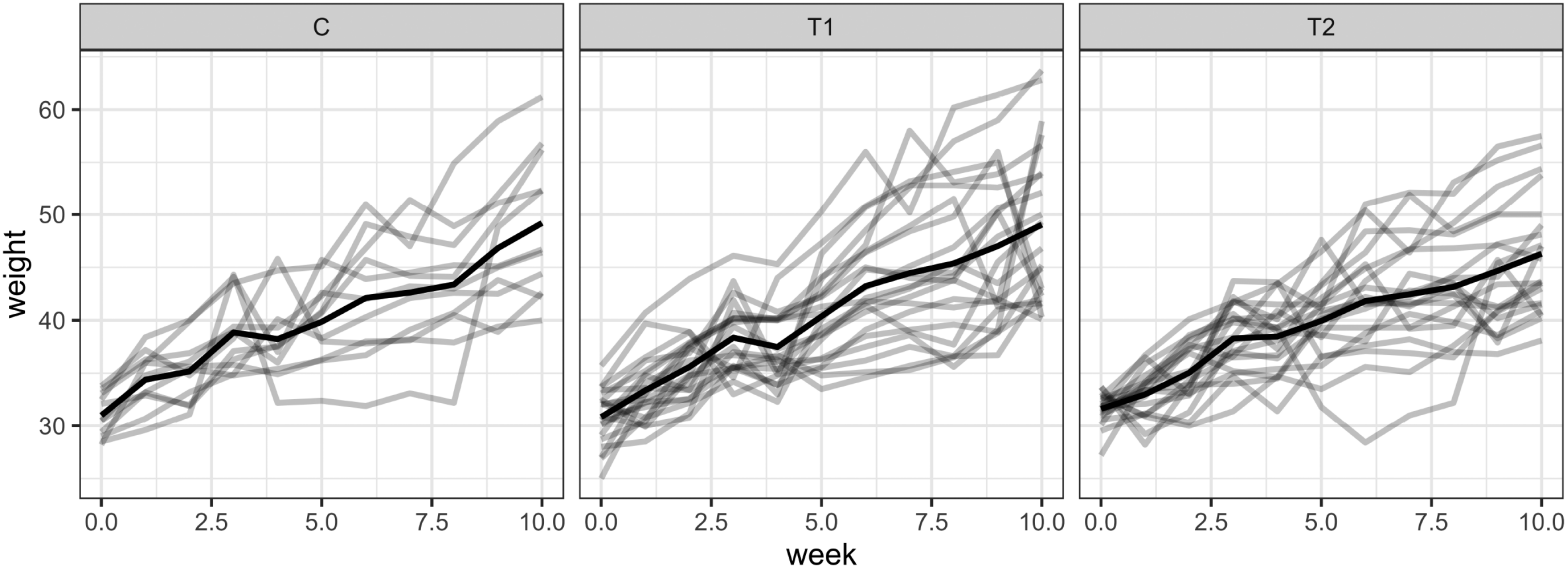
Mice weight data. Individual profiles of the mice's weights on different diets. The highlighted line represents the mean profile.

Following Schumacher et al. [28], we consider an AR(1)‐SSL‐LMM as follows:

(15) $$\begin{array}{ll} Y_{ij} & =(\beta_{0}+b_{0i})+(\beta_{1}+b_{1i})x_{ij}+\beta_{2}1_{\left(treat_{i}=T_{1},x_{ij}\geq 0\right)}\\ & \;+\beta_{3}1_{\left(treat_{i}=T_{2},x_{ij}\geq 0\right)}+\beta_{4}1_{\left(treat_{i}=T_{1},x_{ij}\geq 0\right)}x_{ij}\\ & \;+\beta_{5}1_{\left(treat_{i}=T_{2},x_{ij}\geq 0\right)}x_{ij}+\epsilon_{ij}, \end{array}$$

i=1,…,52, j=1,…,11, where $Y_{ij}$ represents the weight of the ith mouse in the jth week, $x_{ij}$ is the week since the start of the intervention, and $1_{A}$ denotes an indicator function that assumes the value 1 when A is true, and 0 otherwise. A more detailed analysis of the model choice can be found in Schumacher et al. [28].

To compare the standard error estimates obtained by the AVE, bootstrap, and SVE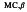 methods for the fixed effects of the model fitted to the mice weight dataset, Table 7 presents the 95% confidence intervals and their respective lengths. The bootstrap and SVE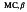 methods were applied on the basis of 100 simulated samples. It is observed that the intervals obtained by the bootstrap and SVE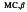 methods are narrower than the interval obtained by AVE, indicating that the standard error estimates provided by these two methods are smaller. Furthermore, when testing the null hypothesis that the interaction between treatment 2 and time is not significant (i.e., $H_{0}:\beta_{5}=0$), the SVE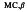, and bootstrap methods lead to similar conclusions. In contrast, the asymptotic method points to a divergent result. As observed in the simulation studies, in small to moderate sample sizes, the asymptotic method can overestimate the standard error, leading to conservative confidence intervals.

**TABLE 7:** Mice weight data.

		AVE		Bootstrap		SVE 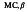		Interval length		
Parameter	Estimate	2.5%	97.5%	2.5%	97.5%	2.5%	97.5%	AVE	bootstrap	SVE
$\beta_{0}$	39.863	38.438	41.288	38.625	41.077	39.092	40.633	2.850	2.452	1.541
$\beta_{1}$	2.035	1.673	2.396	1.741	2.294	1.809	2.261	0.723	0.553	0.452
$\beta_{2}$	−2.171	−4.218	−0.125	−3.195	−1.077	−3.257	−1.086	4.093	2.118	2.171
$\beta_{3}$	−0.854	−2.623	0.916	−1.858	0.141	−1.962	0.255	3.539	1.998	2.217
$\beta_{4}$	−0.169	−0.612	0.275	−0.551	0.206	−0.541	0.204	0.887	0.757	0.745
$\beta_{5}$	−0.470	−1.015	0.075	−0.847	−0.127	−0.846	−0.094	1.090	0.721	0.752

*Note:* 95% confidence intervals and their lengths for the fixed effects of the AR(1)‐SSL model fit, obtained using the asymptotic variance estimator (AVE), bootstrap, and sandwich variance estimator (SVE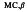) methods.

To evaluate the results obtained considering AR(1)‐SSL model in comparison with those from the N, SN, and ST models under an AR(1) dependence structure, as well as the SSL‐LMM with UNC and DEC dependence structures, Figure 3 shows the 95% confidence intervals for the fixed effects, obtained using the AVE, bootstrap, and SVE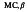 methods. The parameters $\beta_{0}$, $\beta_{1}$, and $\beta_{2}$ showed statistical significance across all models and methods used to construct the confidence intervals, as none of their 95% confidence intervals contain zero. On the other hand, the parameter $\beta_{3}$ is not statistically significant in most cases, except when using the bootstrap method under the N‐AR(1) model. The significance of $\beta_{4}$ and $\beta_{5}$ depends on the model, dependence structure, and confidence interval estimation method. In addition, the bootstrap and SVE methods lead to similar conclusions regarding the statistical significance of the parameters under the different models. The confidence intervals and their lengths are presented in Table D1 of Appendix D.

**FIGURE 3:**
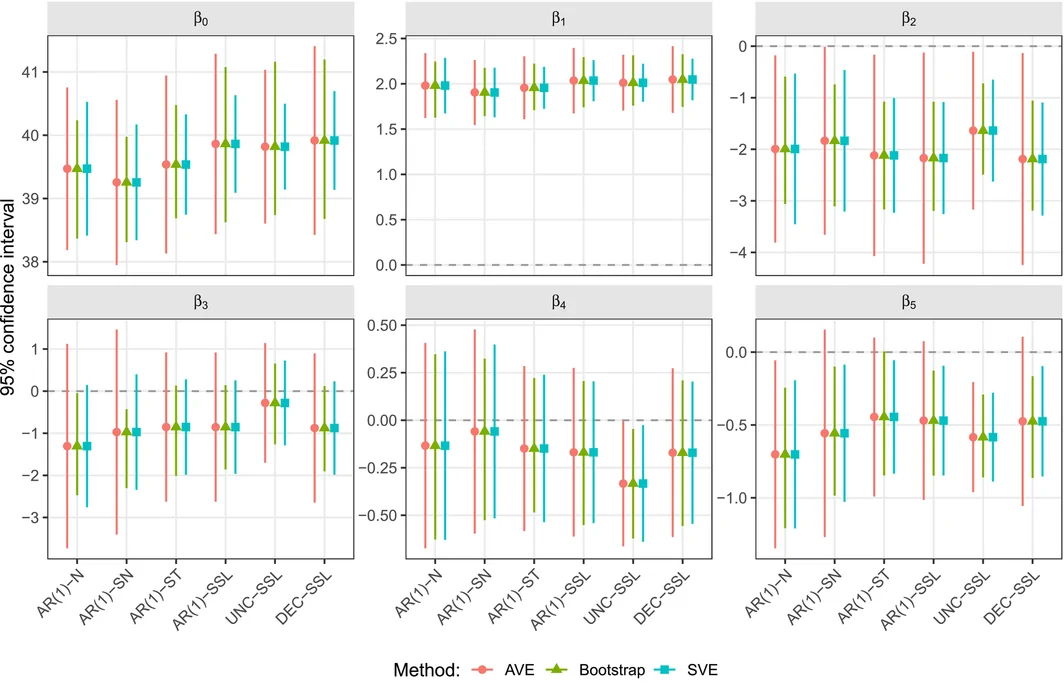
Mice weight data. 95% confidence intervals for the fixed effects, obtained using the AVE, bootstrap, and SVE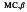 methods considering different models.

Figure 4 shows the Mahalanobis distance for the mice weight data, considering the Normal and SSL models with an AR(1) dependence structure for the error covariance matrix. It can be observed that for the AR(1)‐SSL‐LMM, all distances are below the theoretical 99% quantile (represented by the dashed blue line), whereas for the AR(1)‐N‐LMM, we observe subjects with distances greater than the given quantile. This behavior highlights the robustness of the SSL distribution in accommodating atypical observations.

**FIGURE 4:**
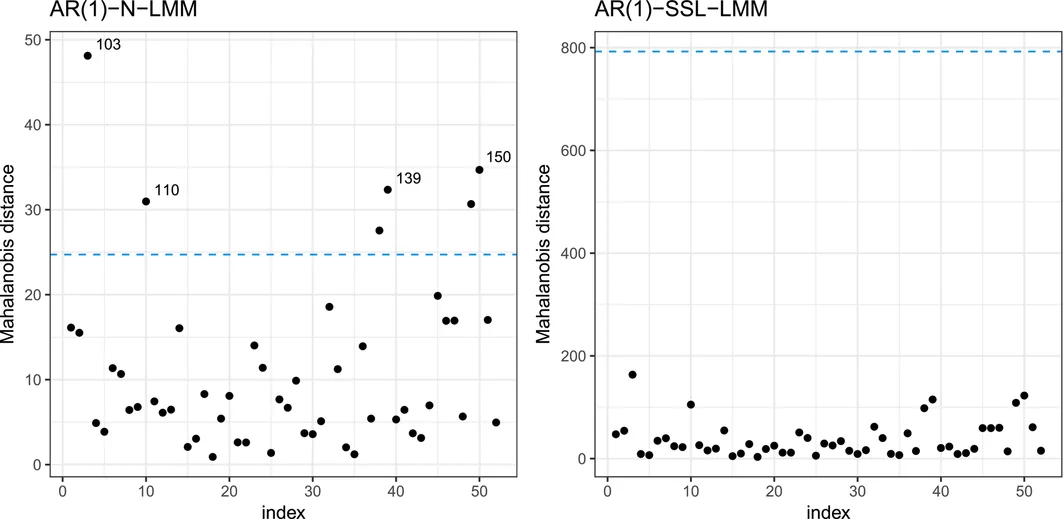
Mahalanobis distance for the mice weight data. The dashed blue line represents the theoretical 99% quantile.

The computational execution time of the methods presents significant differences. The AVE method had the shortest execution time, with obtaining the 95% confidence interval taking less than 0.001 min. The bootstrap method took 9.96 min, while SVE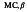 took 1.99 min.

## 5 Conclusions

Considering that the estimation of the standard error is a fundamental aspect in the analysis of mixed models, as it directly influences, for example, the inferences made about the fixed effects, in this work, we present the use of the sandwich variance estimator for scale mixture of SMSN‐LMM with serial dependence within the individual.

Based on the simulation studies, the AVE method presents computational simplicity and, for large sample sizes, the estimates for fixed effects converge to those of the MC‐SD, bootstrap, and SVE methods. However, as demonstrated in the results, it can present an overestimation of the standard error, especially under misspecification or under small samples. The bootstrap, in turn, allows a more flexible estimation of variability, being less dependent on model assumptions. However, its main limitation is the high computational cost, especially in complex models or datasets with many subjects.

The SVE method for fixed effects, which are usually the primary focus of inference in practical applications, stands out for its robustness and good approximation to the standard error obtained using the Monte Carlo method, even in scenarios with a small number of subjects and misspecification of the random effects distribution or the correlation structure of errors. However, as observed in the simulation studies, for parameters associated with the distribution of the random effects such as the skewness and the covariance matrix, the SVE did not provide reliable standard error estimates. Consequently, while the SVE remains a fast and robust method of choice for inference on fixed effects under model misspecification on small to moderate sample sizes, bootstrap methods are recommended when the main objective is to estimate the standard error of the parameters of the random effects structure. Furthermore, adequate specification of the distribution of random effects and the dependence structure of errors is essential to ensure the validity of inferential conclusions.

The proposed SVE was developed for the SN, skew‐t (ST), SSL, and SCN distributions, as well as their symmetric versions, under UNC, DEC, and AR(p) dependency structures. It was incorporated into the confint function of the R package **skewlmm** [28], available in the comprehensive R archive network (CRAN) repository, and can be obtained using the method = “sandwich” argument.

## Funding

This work was supported by the Fundação de Amparo à Pesquisa do Estado de São Paulo (Grant No. 2020/16713‐0), the Conselho Nacional de Desenvolvimento Científico e Tecnológico (Grant No. 307105/2022‐9), the Coordenação de Aperfeiçoamento de Pessoal de Nível Superior (Grant No. 001), and by L'ORÉAL‐UNESCO‐ABC Para Mulheres na Ciência.

## Conflicts of Interest

The authors declare no conflicts of interest.

## Data Availability

The data that support the findings of this study are openly available in R package skewlmm at https://CRAN.R‐project.org/package=skewlmm.

## Appendix A Derivatives of Hessian Matrix

The derivatives necessary to obtain the Hessian matrix in (6) are presented below. Let ${\dot{F}}_{k}=\partial F/\partial \alpha_{k}$, r=1,…,q(q+1)/2, ${\dot{R}}_{ir}=\partial R_{i}/\partial \phi_{r}$, and ${\ddot{R}}_{ist}=\partial^{2}R_{i}/\partial \phi_{r}\partial \phi_{s}$, s,t=1,…,p. The derivatives of $R_{i}$ depend on the dependency structure used. Lachos et al. [29] present the derivatives for the case where R is a DEC matrix. For the case where R is a dependency matrix AR(p), in which the expressions for the autocorrelation matrix vary according to the value of p, it is necessary to use numerical derivatives of the theoretical autocorrelation function of the AR process. These derivatives can be calculated in R software using the ARMAacf() function.

• For $\log|\Psi_{i}|$, we obtain,

$$\begin{array}{cc} \frac{\partial^{2}\log|\Psi_{i}|}{\partial \tau_{1}\partial \tau_{1}^{⊤}} & =0,\;\text{for}\;\tau_{1}=\beta ,\lambda \;\text{and}\;\nu ,\\ \frac{\partial^{2}\log|\Psi_{i}|}{\partial \tau_{1}\partial \tau_{2}^{⊤}} & =0,\;\text{for}\;\tau_{2}=\sigma_{e}^{2},\alpha_{k}\;\text{and}\;\phi_{r},\\ \frac{\partial^{2}\log|\Psi_{i}|}{\partial \sigma_{e}^{2}\partial \sigma_{e}^{2}} & =-\;\text{tr}\left(\Psi_{i}^{-1}R_{i}\Psi_{i}^{-1}R_{i}\right),\\ \frac{\partial^{2}\log|\Psi_{i}|}{\partial \sigma_{e}^{2}\partial \phi_{r}} & =\text{tr}\left(\Psi_{i}^{-1}{\dot{R}}_{ir}\left(I-\sigma_{e}^{2}\Psi_{i}^{-1}R_{i}\right)\right),\\ \frac{\partial^{2}\log|\Psi_{i}|}{\partial \alpha_{k}\partial \alpha_{l}} & =\text{tr}\left(D_{\left(k,l\right)}^{1}-D_{\left(l,0\right)}^{1}D_{\left(k,0\right)}^{1}\right),\\ \frac{\partial^{2}\log|\Psi_{i}|}{\partial \alpha_{k}\partial \phi_{r}} & =-\;\text{tr}\left(\sigma_{e}^{2}\Psi_{i}^{-1}{\dot{R}}_{ir}D_{\left(k,0\right)}^{1}\right),\\ \frac{\partial^{2}\log|\Psi_{i}|}{\partial \phi_{r}\partial \phi_{s}} & =\sigma_{e}^{2}\;\text{tr}\left(\Psi_{i}^{-1}{\ddot{R}}_{ist}-\sigma_{e}^{2}\Psi_{i}^{-1}{\dot{R}}_{it}\Psi_{i}^{-1}{\dot{R}}_{ir}\right), \end{array}$$

for r=1,…,q(q+1)/2 and s,t=1,…,p; where $D_{\left(x,y\right)}^{1}=\Psi_{i}^{-1}Z_{i}({\dot{F}}_{x}{\dot{F}}_{y}+{\dot{F}}_{y}{\dot{F}}_{x})Z_{i}^{⊤}$, with ${\dot{F}}_{0}=F$.

• For $A_{i}$, we obtain,

$$\begin{array}{ll} \frac{\partial^{2}A_{i}}{\partial \beta \partial \beta^{⊤}} & =0,\\ \frac{\partial^{2}A_{i}}{\partial \beta \partial \sigma_{e}^{2}} & =\frac{1}{a_{i}}X_{i}^{⊤}D_{\left(0,0\right)}^{3^{⊤}}\lambda +\frac{1}{2a_{i}^{3}\sigma_{e}^{4}}X_{i}^{⊤}\Psi_{i}^{-1}Z_{i}F\lambda \lambda^{⊤}D_{\left(0\right)}^{2}\Lambda_{i}F^{-1}\lambda ,\\ \frac{\partial^{2}A_{i}}{\partial \beta \partial \phi_{r}} & =\frac{\sigma_{e}^{2}}{a_{i}}X_{i}^{⊤}D_{\left(0,r\right)}^{3^{⊤}}\lambda +\frac{1}{2a_{i}^{3}\sigma_{e}^{2}}X_{i}^{⊤}\Psi_{i}^{-1}Z_{i}F\lambda \lambda^{⊤}D_{\left(r\right)}^{2}\Lambda_{i}F^{-1}\lambda ,\\ \frac{\partial^{2}A_{i}}{\partial \beta \partial \alpha_{k}} & =\frac{1}{a_{i}}X_{i}^{⊤}D_{\left(0,k\right)}^{1}\Psi_{i}^{-1}Z_{i}F\lambda -\frac{1}{a_{i}}X_{i}^{⊤}\Psi_{i}^{-1}Z_{i}{\dot{F}}_{k}\lambda \\ & \;-\frac{1}{2a_{i}^{3}}X_{i}^{⊤}\Psi_{i}^{-1}Z_{i}F\lambda \lambda^{⊤}F^{-1}D_{\left(k\right)}^{4}\lambda ,\\ \frac{\partial^{2}A_{i}}{\partial \beta \partial \lambda^{⊤}} & =-\frac{1}{a_{i}}X_{i}^{⊤}\Psi_{i}^{-1}Z_{i}F+\frac{1}{a_{i}^{3}}X_{i}^{⊤}\Psi_{i}^{-1}Z_{i}F\lambda \lambda^{⊤}F^{-1}\Lambda_{i}F^{-1},\\ \frac{\partial^{2}A_{i}}{\partial \sigma_{e}^{2}\partial \sigma_{e}^{2}} & =\frac{2}{a_{i}}\lambda^{⊤}D_{\left(0,0\right)}^{3}R_{i}\Psi_{i}^{-1}(y_{i}-X_{i}\beta -cZ_{i}\Delta )\\ & \;+\frac{1}{2a_{i}^{3}\sigma_{e}^{4}}\lambda^{⊤}D_{\left(0,0\right)}^{3}(y_{i}-X_{i}\beta -cZ_{i}\Delta )\lambda^{⊤}D_{\left(0\right)}^{2}\Lambda_{i}F^{-1}\lambda \\ & \;-\frac{1}{2a_{i}^{2}\sigma_{e}^{4}}{\dot{A}}_{i\sigma_{e}^{2}}\lambda^{⊤}D_{\left(0\right)}^{2}\Lambda_{i}F^{-1}\lambda \\ & \;\;\;\;-\frac{1}{a_{i}^{2}\sigma_{e}^{8}}A_{i}\lambda^{⊤}D_{\left(0\right)}^{2}\Lambda_{i}D_{\left(0\right)}^{2^{⊤}}\lambda +\frac{1}{a_{i}^{2}\sigma_{e}^{6}}A_{i}\lambda^{⊤}D_{\left(0\right)}^{2}\Lambda_{i}F^{-1}\lambda ,\\ \frac{\partial^{2}A_{i}}{\partial \sigma_{e}^{2}\partial \phi_{r}} & =-\frac{1}{a_{i}}\lambda^{⊤}D_{\left(0,r\right)}^{3}(y_{i}-X_{i}\beta -cZ_{i}\Delta )\\ & \;+\frac{2\sigma_{e}^{2}}{a_{i}}\lambda^{⊤}D_{\left(0,r\right)}^{3}R_{i}\Psi_{i}^{-1}(y_{i}-X_{i}\beta -cZ_{i}\Delta )\\ & \;\;\;+\frac{1}{2a_{i}^{3}\sigma_{e}^{2}}\lambda^{⊤}D_{\left(0,0\right)}^{3}(y_{i}-X_{i}\beta -cZ_{i}\Delta )\lambda^{⊤}D_{\left(r\right)}^{2}\Lambda_{i}F^{-1}\lambda \\ & \;-\frac{1}{2a_{i}^{2}\sigma_{e}^{4}}{\dot{A}}_{i\phi_{r}}\lambda^{⊤}D_{\left(0\right)}^{2}\Lambda_{i}F^{-1}\lambda -\frac{1}{a_{i}^{2}\sigma_{e}^{6}}A_{i}\lambda^{⊤}D_{\left(r\right)}^{2}\Lambda_{i}D_{\left(0\right)}^{2^{⊤}}\lambda \\ & \;\;\;+\frac{1}{2a_{i}\sigma_{e}^{4}}A_{i}\lambda^{⊤}D_{\left(r\right)}^{2}\Lambda_{i}F^{-1}\lambda \\ & \;+\frac{1}{2a_{i}^{4}\sigma_{e}^{6}}A_{i}\lambda^{⊤}D_{\left(0\right)}^{2}\Lambda F^{-1}\lambda \lambda^{⊤}D_{\left(r\right)}^{2}\Lambda_{i}F^{-1}\lambda ,\\ \frac{\partial^{2}A_{i}}{\partial \sigma_{e}^{2}\partial \alpha_{k}} & =-\frac{1}{a_{i}}\lambda^{⊤}D_{\left(k,0\right)}^{3}(y_{i}-X_{i}\beta -cZ_{i}\Delta )\\ & \;+\frac{1}{a_{i}}\lambda^{⊤}FZ_{i}^{⊤}D_{\left(0,k\right)}^{1}\Psi_{i}^{-1}R_{i}\Psi_{i}^{-1}(y_{i}-X_{i}\beta -cZ_{i}\Delta ),\\ \frac{\partial^{2}A_{i}}{\partial \sigma_{e}^{2}\partial \lambda } & =-\frac{1}{a_{i}}D_{\left(0,0\right)}^{3}(y_{i}-X_{i}\beta -cZ_{i}\Delta )+\frac{c}{a_{i}}\lambda^{⊤}D_{\left(0,0\right)}^{3}Z_{i}FD^{6}\\ & \;+\frac{1}{a_{i}^{3}}\lambda^{⊤}D_{\left(0,0\right)}^{3}(y_{i}-X_{i}\beta -cZ_{i}\Delta )\lambda^{⊤}F^{-1}\Lambda_{i}F^{-1}\\ & \;\;\;\;-\frac{1}{2\sigma_{e}^{4}a_{i}^{2}}({\dot{A}}_{i\lambda }^{⊤}\lambda^{⊤}F^{-1}\Lambda_{i}Z_{i}^{⊤}\Psi_{i}^{-1}R_{i}Z_{i}\Lambda_{i}F^{-1}\lambda \\ & \;+2A_{i}\lambda^{⊤}F^{-1}\Lambda_{i}Z_{i}^{⊤}\Psi_{i}^{-1}R_{i}Z_{i}\Lambda_{i}F^{-1}),\\ \frac{\partial^{2}A_{i}}{\partial \phi_{r}\partial \phi_{s}} & =-\frac{\sigma_{e}^{2}}{a_{i}}\lambda^{⊤}FZ_{i}^{⊤}\Psi_{i}^{-1}({\ddot{R}}_{irs}-\sigma_{e}^{2}{\dot{R}}_{is}\Psi_{i}^{-1}{\dot{R}}_{ir}\\ & \;+\sigma_{e}^{2}{\dot{R}}_{ir}\Psi_{i}^{-1}{\dot{R}}_{is})\Psi_{i}^{-1}(y_{i}-X_{i}\beta -cZ_{i}\Delta )\\ & \;\;\;+\frac{1}{2a_{i}^{3}}\lambda^{⊤}D_{\left(0,r\right)}^{3}(y_{i}-X_{i}\beta -cZ_{i}\Delta )\lambda^{⊤}D_{\left(s\right)}^{2}\Lambda_{i}F^{-1}\lambda \\ & \;-\frac{1}{2a_{i}^{2}\sigma_{e}^{2}}{\dot{A}}_{i\phi_{s}}\lambda^{⊤}D_{\left(r\right)}^{2}\Lambda_{i}F^{-1}\lambda -\frac{1}{2a_{i}^{2}\sigma_{e}^{4}}A_{i}\lambda^{⊤}D_{\left(s\right)}^{2}\Lambda_{i}D_{\left(r\right)}^{2^{⊤}}\lambda \\ & \;\;\;\;-\frac{1}{2a_{i}^{2}\sigma_{e}^{2}}A_{i}\lambda^{⊤}F^{-1}\Lambda_{i}Z_{i}^{⊤}R_{i}^{-1}({\ddot{R}}_{irs}-{\dot{R}}_{is}R_{i}^{-1}{\dot{R}}_{ir}\\ & \;-{\dot{R}}_{ir}R_{i}^{-1}{\dot{R}}_{is})R_{i}^{-1}Z_{i}\Lambda_{i}F^{-1}\lambda \\ & \;\;\;\;-\frac{1}{2a_{i}^{2}\sigma_{e}^{4}}A_{i}\lambda^{⊤}D_{\left(r\right)}^{2}\Lambda_{i}D_{\left(s\right)}^{2^{⊤}}\lambda \\ & \;+\frac{1}{2a_{i}^{4}\sigma_{e}^{4}}A_{i}\lambda^{⊤}D_{\left(r\right)}^{2}\Lambda_{i}F^{-1}\lambda \lambda^{⊤}D_{\left(s\right)}^{2}\Lambda_{i}F^{-1}\lambda ,\\ \frac{\partial^{2}A_{i}}{\partial \phi_{r}\partial \alpha_{k}} & =-\frac{\sigma_{e}^{2}}{a_{i}}\lambda^{⊤}D_{\left(k,r\right)}^{3}(y_{i}-X_{i}\beta -cZ_{i}\Delta )+\frac{\sigma_{e}^{2}}{a_{i}}\lambda^{⊤}FZ_{i}^{⊤}D_{\left(k,0\right)}^{1}\\ & \;\times \Psi_{i}^{-1}{\dot{R}}_{ir}\Psi_{i}^{-1}(y_{i}-X_{i}\beta -cZ_{i}\Delta )+\frac{c\sigma_{e}^{2}}{a_{i}}\lambda^{⊤}D_{\left(0,r\right)}^{3}Z_{i}{\dot{F}}_{k}\delta \\ & \;\;\;+\frac{\sigma_{e}^{2}}{a_{i}}\lambda^{⊤}FZ_{i}^{⊤}\Psi_{i}^{-1}{\dot{R}}_{ir}D_{\left(k,0\right)}^{1}\Psi_{i}^{-1}(y_{i}-X_{i}\beta -cZ_{i}\Delta )\\ & \;-\frac{\sigma_{e}^{2}}{2a_{i}^{3}}\lambda^{⊤}D_{\left(0,r\right)}^{3}(y_{i}-X_{i}\beta -cZ_{i}\Delta )\lambda^{⊤}F^{-1}D_{\left(k\right)}^{4}\lambda \\ & \;\;\;\;-\frac{1}{2a_{i}^{2}\sigma_{e}^{2}}({\dot{A}}_{i\alpha_{k}}\lambda^{⊤}-A_{i}\lambda^{⊤}F^{-1}{\dot{F}}_{k})D_{\left(r\right)}^{2}\Lambda_{i}F^{-1}\lambda \\ & \;-\frac{1}{2a_{i}^{2}\sigma_{e}^{2}}A_{i}\lambda^{⊤}F^{-1}\Lambda_{i}D_{\left(k,0\right)}^{5}\Lambda_{i}D_{\left(r\right)}^{2^{⊤}}\lambda \\ & \;\;\;\;-\frac{1}{2a_{i}^{2}\sigma_{e}^{2}}A_{i}\lambda^{⊤}D_{\left(r\right)}^{2}\Lambda_{i}D_{\left(k,0\right)}^{5}\Lambda_{i}F^{-1}\lambda \\ & \;+\frac{1}{2a_{i}^{2}\sigma_{e}^{2}}A_{i}\lambda^{⊤}D_{\left(r\right)}^{2}\Lambda_{i}F^{-1}{\dot{F}}_{k}F^{-1}\lambda \\ & \;+\frac{1}{2a_{i}^{4}\sigma_{e}^{2}}A_{i}\lambda^{⊤}D_{\left(r\right)}^{2}\Lambda_{i}F^{-1}\lambda \lambda^{⊤}F^{-1}D_{\left(k\right)}^{4}\lambda ,\\ \frac{\partial^{2}A_{i}}{\partial \phi_{r}\partial \lambda } & =-\frac{\sigma_{e}^{2}}{a_{i}}D_{\left(0,r\right)}^{3}(y_{i}-X_{i}\beta -cZ_{i}\Delta )+\frac{c\sigma_{e}^{2}}{a_{i}}\lambda^{⊤}D_{\left(0,r\right)}^{3}Z_{i}^{⊤}FD^{6}\\ & \;\times +\frac{\sigma_{e}^{2}}{a_{i}^{3}}\lambda^{⊤}D_{\left(0,r\right)}^{3}(y_{i}-X_{i}\beta -cZ_{i}\Delta )F^{-1}\Lambda_{i}F^{-1}\lambda \\ & \;\;\;\;-\frac{1}{2a_{i}^{2}\sigma_{e}^{2}}({\dot{A}}_{i\lambda }\lambda^{⊤}+2A_{i})D_{\left(r\right)}^{2}\Lambda_{i}F^{-1}\lambda \\ & \;\times -\frac{1}{a_{i}^{4}\sigma_{e}^{2}}A_{i}D_{\left(r\right)}^{2}\Lambda_{i}F^{-1}\lambda F^{-1}\Lambda_{i}F^{-1}\lambda ,\\ \frac{\partial^{2}A_{i}}{\partial \alpha_{k}\partial \alpha_{l}} & =-\frac{1}{a_{i}}\lambda^{⊤}\left({\dot{F}}_{k}Z_{i}^{⊤}D_{\left(0,l\right)}^{1}+{\dot{F}}_{l}Z_{i}^{⊤}D_{\left(0,k\right)}^{1}-FZ_{i}^{⊤}\left(D_{\left(0,l\right)}^{1}D_{\left(0,k\right)}^{1}\right.\right.\\ & \;\left.\left.+D_{\left(0,k\right)}^{1}D_{\left(0,l\right)}^{1}-D_{\left(k,l\right)}^{1}\right)\right)\Psi^{-1}(y_{i}-X_{i}\beta -cZ_{i}\Delta )\\ & \;\;\;\;-\frac{c}{a_{i}}\left(\lambda^{⊤}{\dot{F}}_{k}Z_{i}^{⊤}\Psi^{-1}Z_{i}{\dot{F}}_{l}\delta +\delta^{⊤}{\dot{F}}_{l}Z_{i}^{⊤}\Psi^{-1}Z_{i}{\dot{F}}_{k}\lambda \right.\\ & \;\left.-\Delta^{⊤}Z_{i}^{⊤}D_{\left(0,l\right)}^{1}\Psi^{-1}Z_{i}{\dot{F}}_{k}\lambda \right)+\frac{1}{a_{i}}\lambda^{⊤}FZ_{i}^{⊤}D_{\left(0,k\right)}^{1}\Psi^{-1}Z_{i}{\dot{F}}_{l}\delta \\ & \;\;\;+\frac{1}{2a_{i}^{2}}A_{i}\lambda^{⊤}F^{-1}\left(-{\dot{F}}_{k}F^{-1}{\dot{F}}_{l}F^{-1}\Lambda_{i}+{\dot{F}}_{k}F^{-1}\Lambda_{i}D_{\left(l,0\right)}^{5}\Lambda_{i}\right.\\ & \;+\Lambda_{i}D_{\left(l,0\right)}^{5}\Lambda_{i}F^{-1}{\dot{F}}_{k}-\Lambda_{i}F^{-1}{\dot{F}}_{l}F^{-1}{\dot{F}}_{k}+\Lambda_{i}D_{\left(k,0\right)}^{5}{\dot{F}}_{l}F^{-1}\Lambda_{i}.\\ & \;+\Lambda_{i}D_{\left(k,l\right)}^{5}\Lambda_{i}-\Lambda_{i}D_{\left(k,0\right)}^{5}\Lambda_{i}D_{\left(l,0\right)}^{5}\Lambda_{i}-(\Lambda_{i}D_{\left(l,0\right)}^{5}\Lambda_{i}F^{-1}\\ & \;\left.-\Lambda_{i}F^{-1}{\dot{F}}_{l}F^{-1})(F^{-1}{\dot{F}}_{k}+{\dot{F}}_{k}F^{-1})F^{-1}\Lambda_{i}\right)F^{-1}\lambda \\ & \;\;\;\;-\frac{1}{2a_{i}^{2}}A_{i}\lambda^{⊤}D_{\left(k\right)}^{4^{⊤}}F^{-1}{\dot{F}}_{l}F^{-1}\lambda +\frac{1}{2a_{i}^{2}}({\dot{A}}_{i\alpha_{l}}\lambda^{⊤}F^{-1}\\ & \;-A_{i}\lambda^{⊤}F^{-1}{\dot{F}}_{l}F^{-1})D_{\left(k\right)}^{4}\lambda -\frac{c}{2a_{i}^{3}}\Delta^{⊤}Z_{i}^{⊤}\Psi^{-1}Z_{i}{\dot{F}}_{k}\lambda \lambda^{⊤}\\ & \;\times F^{-1}D_{\left(l\right)}^{4}\lambda \\ & \;\;\;\;-\frac{1}{a_{i}^{3}}\lambda^{⊤}\left(\frac{1}{2}{\dot{F}}_{k}Z_{i}^{⊤}+FZ_{i}^{⊤}D_{\left(0,k\right)}^{1}\right)\Psi^{-1}(y_{i}-X_{i}\beta -cZ_{i}\Delta )\lambda^{⊤}\\ & \;\times F^{-1}D_{\left(l\right)}^{4}\lambda +\frac{1}{2a_{i}^{4}}A_{i}\lambda^{⊤}D_{\left(k\right)}^{4^{⊤}}F^{-1}\lambda \lambda^{⊤}F^{-1}D_{\left(l\right)}^{4}\lambda ,\\ \frac{\partial^{2}A_{i}}{\partial \alpha_{k}\partial \lambda } & =\frac{1}{a_{i}}{\left(y_{i}-X_{i}\beta -cZ_{i}\Delta \right)}^{⊤}\Psi_{i}^{-1}Z_{i}{\dot{F}}_{k}\\ & \;-\frac{c}{a_{i}}\lambda^{⊤}{\dot{F}}_{k}Z_{i}^{⊤}\Psi_{i}^{-1}Z_{i}FD^{6}-\frac{c}{a_{i}}D^{6}FZ_{i}^{⊤}\Psi_{i}^{-1}Z_{i}{\dot{F}}_{k}\lambda \\ & \;+\frac{1}{2a_{i}^{2}}({\dot{A}}_{i\lambda }\lambda^{⊤}+A_{i})F^{-1}D_{\left(k\right)}^{4}\lambda \\ & \;\;\;\;-\frac{1}{a_{i}^{3}}\lambda^{⊤}{\dot{F}}_{k}Z_{i}^{⊤}\Psi_{i}^{-1}(y_{i}-X_{i}\beta -cZ_{i}\Delta )\lambda^{⊤}F^{-1}\Lambda_{i}F^{-1}\\ & \;-\frac{c}{a_{i}^{3}}\Delta^{⊤}Z_{i}^{⊤}\Psi_{i}^{-1}Z_{i}{\dot{F}}_{k}(a_{i}^{2}+\lambda \lambda^{⊤}F^{-1}\Lambda_{i}F^{-1})\\ & \;\;\;\;-\frac{1}{a_{i}^{3}}\lambda^{⊤}FZ_{i}^{⊤}D_{\left(0,k\right)}^{1}\Psi_{i}^{-1}(y_{i}-X_{i}\beta -cZ_{i}\Delta )\lambda^{⊤}F^{-1}\Lambda_{i}F^{-1}\\ & \;+\frac{1}{2a_{i}^{4}}A_{i}\lambda^{⊤}F^{-1}D_{\left(k\right)}^{4}(a_{i}^{2}-2\lambda \lambda^{⊤}F^{-1}\Lambda_{i}F^{-1}),\\ \frac{\partial^{2}A_{i}}{\partial \lambda \partial \lambda } & =-\frac{2c}{a_{i}}FZ_{i}^{⊤}\Psi_{i}^{-1}Z_{i}FD^{6}-\frac{1}{a_{i}^{3}}FZ_{i}^{⊤}\Psi_{i}^{-1}(y_{i}-X_{i}\beta -cZ_{i}\Delta )\lambda^{⊤}\\ & \;\times F^{-1}\Lambda_{i}+\Lambda_{i}F^{-1}+\frac{2c}{a_{i}(1+\lambda^{⊤}\lambda )}FZ_{i}^{⊤}\Psi_{i}^{-1}Z_{i}F\lambda \delta \\ & \;\;\;+\frac{c}{a_{i}(1+\lambda^{⊤}\lambda )}\lambda^{⊤}FZ_{i}^{⊤}\Psi_{i}^{-1}Z_{i}F\lambda D^{6}\\ & \;-\frac{1}{a_{i}^{2}}{\dot{A}}_{i\lambda }F^{-1}\Lambda_{i}F^{-1}\lambda^{⊤}\\ & \;-\frac{1}{a_{i}^{4}}A_{i}F^{-1}\Lambda_{i}F^{-1}(a_{i}^{2}-2\lambda^{⊤}\lambda F^{-1}\Lambda_{i}F^{-1})\\ & \;\;\;\;-\frac{c}{a_{i}^{2}{\left(1+\lambda^{⊤}\lambda \right)}^{5/2}}\lambda^{⊤}FZ_{i}^{⊤}\Psi_{i}^{-1}Z_{i}F\lambda \lambda^{⊤}\\ & \;\times \left(\frac{1}{a_{i}}\lambda^{⊤}F^{-1}\Lambda_{i}F^{-1}(1+\lambda^{⊤}\lambda )+2a_{i}\lambda^{⊤}\right), \end{array}$$

where $a_{i}={\left(1+\lambda F^{-1}\Lambda_{i}F^{-1}\lambda \right)}^{1/2}$, $\Lambda_{i}=D^{-1}+Z_{i}^{⊤}\Sigma_{i}^{-1}Z_{i}$, ${\dot{A}}_{i\sigma_{e}^{2}}=\partial A_{i}/\partial \sigma_{e}^{2}$, ${\dot{A}}_{i\phi_{r}}=\partial A_{i}/\partial \phi_{r}$, ${\dot{A}}_{i\alpha_{k}}=\partial A_{i}/\partial \alpha_{k}$, ${\dot{A}}_{i\lambda }=\partial A_{i}/\partial \lambda$. Also, $D_{\left(x\right)}^{2}=F^{-1}\Lambda_{i}Z_{i}^{⊤}R_{i}^{-1}{\dot{R}}_{ix}R_{i}^{-1}Z_{i}$, $D_{\left(x,y\right)}^{3}={\dot{F}}_{x}Z_{i}^{⊤}\Psi_{i}^{-1}{\dot{R}}_{iy}\Psi_{i}^{-1}$, $D_{\left(x\right)}^{4}=({\dot{F}}_{x}F^{-1}\Lambda_{i}+\Lambda_{i}F^{-1}{\dot{F}}_{x}-\Lambda_{i}F^{-1}({\dot{F}}_{x}F^{-1}+F^{-1}{\dot{F}}_{x})F^{-1}\Lambda_{i})F^{-1}$, $D_{\left(x,y\right)}^{5}=F^{-1}({\dot{F}}_{x}F^{-1}{\dot{F}}_{y}F^{-1}+F^{-1}{\dot{F}}_{y}F^{-1}{\dot{F}}_{x})F^{-1}$ and $D^{6}={\left(\frac{(1+\lambda^{⊤}\lambda )I_{q}-\lambda \lambda^{⊤}}{{\left(1+\lambda^{⊤}\lambda \right)}^{3/2}}\right)}^{⊤}$, with ${\dot{R}}_{i0}=R_{i}$.

• And, for $d_{i}$ we have,

## Appendix B Additional Results for Simulation Study 1

Table A1 presents the mean interval lengths and coverage probabilities for 95% confidence intervals under scenarios (a) and (b) for all parameters of the model presented in (12). The results are based on 500 Monte Carlo samples, obtained using the AVE, bootstrap, and SVE methods, for different numbers of subjects. It is observed that the AVE method presented coverage probabilities closer to the 95% level for almost all sample sizes in both scenarios. The bootstrap method generated narrower intervals; however, for small samples and for the variance and asymmetry parameters, it presented lower coverage probabilities. Conversely, the SVE method demonstrated reasonable coverage probabilities with moderate interval lengths, although it exhibited instability regarding the parameter λ, for which intervals became excessively long in some scenarios. Furthermore, all the methods presented an improvement in performance as the sample size increases.

**TABLE A1:** Simulation Study 1.

		Scenario (a)						Scenario (b)					
		Coverage probabilities			Mean interval lengths			Coverage probabilities			Mean interval lengths		
*n*	Param.	AVE	Boot.	SVE	AVE	Boot.	SVE	AVE	Boot.	SVE	AVE	Boot.	SVE
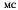
20	$\beta_{0}$	0.980	0.926	0.954	1.197	0.716	0.797	0.982	0.914	0.932	1.219	0.767	0.870
	$\beta_{1}$	0.974	0.904	0.918	2.905	1.952	1.994	0.978	0.940	0.936	2.572	1.790	1.751
	$\beta_{2}$	0.976	0.916	0.946	10.02	6.836	7.316	0.986	0.890	0.938	9.971	6.248	7.245
	$\beta_{3}$	0.986	0.910	0.946	1.536	0.946	1.004	0.978	0.854	0.892	1.605	0.918	1.047
	$\beta_{4}$	0.982	0.908	0.922	3.867	2.590	2.720	0.978	0.924	0.932	3.474	2.284	2.326
	$\sigma_{e}^{2}$	0.948	0.728	0.854	0.201	0.120	0.197	0.940	0.740	0.880	0.203	0.118	0.160
	ϕ	0.984	0.786	0.904	0.868	0.480	1.013	0.982	0.920	0.940	0.850	0.566	0.676
	F	1.000	0.862	0.930	30.742	0.791	3.041	0.990	0.816	0.924	2.629	0.865	1.970
	λ	—	0.596	0.802	—	16.881	75.349	—	0.760	0.914	—	25.933	147.373
50	$\beta_{0}$	0.966	0.930	0.942	0.566	0.469	0.551	0.962	0.946	0.938	0.598	0.549	0.705
	$\beta_{1}$	0.956	0.916	0.930	1.502	1.308	1.343	0.942	0.878	0.906	1.334	1.036	1.155
	$\beta_{2}$	0.948	0.918	0.934	5.196	4.488	4.785	0.946	0.910	0.938	5.134	4.286	4.779
	$\beta_{3}$	0.972	0.916	0.944	0.751	0.613	0.713	0.952	0.896	0.934	0.796	0.625	0.779
	$\beta_{4}$	0.958	0.916	0.932	2.008	1.720	1.793	0.946	0.918	0.926	1.738	1.467	1.519
	$\sigma_{e}^{2}$	0.948	0.840	0.932	0.104	0.083	0.250	0.932	0.854	0.920	0.102	0.077	0.155
	ϕ	0.960	0.856	0.946	0.408	0.306	1.424	0.966	0.916	0.958	0.400	0.327	0.737
	F	1.000	0.870	0.960	3.131	0.620	9.371	0.978	0.842	0.972	0.897	0.576	2.421
	λ	—	0.450	0.772	—	9.856	154.537	—	0.918	0.890	—	15.368	587.413
100	$\beta_{0}$	0.956	0.926	0.952	0.380	0.337	0.382	0.952	0.926	0.958	0.390	0.361	0.418
	$\beta_{1}$	0.932	0.906	0.922	1.019	0.942	1.965	0.962	0.932	0.952	0.879	0.766	0.827
	$\beta_{2}$	0.948	0.922	0.942	3.517	3.235	3.368	0.964	0.958	0.958	3.451	3.338	3.324
	$\beta_{3}$	0.960	0.940	0.952	0.506	0.441	0.470	0.956	0.926	0.940	0.521	0.463	0.480
	$\beta_{4}$	0.944	0.912	0.932	1.350	1.250	1.281	0.952	0.932	0.940	1.151	1.059	1.077
	$\sigma_{e}^{2}$	0.952	0.898	0.944	0.068	0.060	0.095	0.962	0.960	0.964	0.068	0.065	0.081
	ϕ	0.950	0.882	0.958	0.264	0.218	0.472	0.974	0.964	0.972	0.260	0.247	0.343
	F	0.998	0.860	0.974	1.048	0.476	2.059	0.986	0.898	0.996	0.531	0.431	0.992
	λ	—	0.178	0.756	—	6.947	53.499	—	0.978	0.894	—	12.31	157.357
300	$\beta_{0}$	0.944	0.914	0.946	0.210	0.194	0.237	0.948	0.940	0.946	0.220	0.212	0.217
	$\beta_{1}$	0.934	0.918	0.936	0.572	0.542	0.569	0.960	0.912	0.956	0.494	0.426	0.485
	$\beta_{2}$	0.940	0.926	0.940	1.966	1.863	1.949	0.934	0.906	0.934	1.945	1.789	1.924
	$\beta_{3}$	0.948	0.926	0.946	0.278	0.256	0.273	0.950	0.924	0.940	0.288	0.261	0.278
	$\beta_{4}$	0.932	0.914	0.930	0.760	0.725	0.757	0.960	0.898	0.960	0.643	0.529	0.634
	$\sigma_{e}^{2}$	0.940	0.896	0.960	0.038	0.035	0.101	0.956	0.974	0.946	0.038	0.040	0.037
	ϕ	0.958	0.892	0.982	0.145	0.126	0.599	0.940	0.930	0.934	0.142	0.137	0.141
	F	0.984	0.802	0.998	0.410	0.283	2.225	0.956	0.920	0.976	0.274	0.252	0.295
	λ	—	< 0.001	0.692	—	3.892	172.328	—	0.934	0.932	—	6.086	14.839

*Note:* Mean interval lengths and coverage probabilities for 95% confidence intervals under scenarios (a) and (b). The results are based on 500 Monte Carlo samples, obtained considering the methods AVE, SVE, and SVE for different numbers of subjects (n).

Abbreviations: AVE, asymptotic variance estimator; SVE, sandwich variance estimator.

## Appendix C Simulation Study 4

In this simulation, we will evaluate the behavior of the sandwich variance estimator under misspecification of the random effects distribution. For this, we generate 500 Monte Carlo samples of the model presented in (13), considering

$$\begin{array}{ll} b_{i} & \overset{iid.}{∼}{\text{ST}}_{2}\left(\left(\begin{array}{c} -\;1.0405\\ -\;1.2933 \end{array}\right),\left(\begin{array}{cc} 1.5 & 1.3\\ 1.3 & 2.0 \end{array}\right),\left(\begin{array}{c} 10\\ 13 \end{array}\right),5\right)\;\text{and}\;\\ \epsilon_{i} & \overset{ind.}{∼}t_{6}(0,0.25\;{\text{R}}_{i},5). \end{array}$$

The data were fitted using ST‐LMM, SN‐LMM, and T‐LMM models, all with AR(2) structure. Table A2 reports the mean standard error estimated in each of these models based on the Monte Carlo samples, considering the methods AVE and SVE, for different numbers of subjects.

**TABLE A2:** Simulation Study 4.

		Method						
			AVE			SVE		
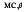
*n*	Parameter	MC‐SD	ST	SN	T	ST	SN	T
20	$\beta_{0}$	0.4226	0.6922	0.9792	0.5334	0.2953	0.3245	0.3501
	$\beta_{1}$	0.7719	1.3379	1.8871	0.9920	0.6089	0.7064	0.6491
	$\beta_{2}$	2.0306	2.6672	3.9315	2.5126	1.6731	1.9623	1.7316
	$\beta_{3}$	0.5690	0.8743	1.2844	0.7587	0.4180	0.4584	0.4954
	$\beta_{4}$	0.9650	1.6214	2.3455	1.3586	0.8234	0.9639	0.8802
50	$\beta_{0}$	0.2763	0.2867	0.3480	0.2638	0.2157	0.2431	0.2355
	$\beta_{1}$	0.4397	0.5401	0.6970	0.4858	0.4126	0.4968	0.4313
	$\beta_{2}$	1.2393	1.2531	1.6755	1.2571	1.1259	1.3098	1.1407
	$\beta_{3}$	0.3518	0.3587	0.4848	0.3666	0.3010	0.3401	0.3292
	$\beta_{4}$	0.5757	0.6782	0.9447	0.6600	0.5682	0.6739	0.5897
100	$\beta_{0}$	0.1751	0.1871	0.2172	0.1788	0.1572	0.1793	0.1682
	$\beta_{1}$	0.3352	0.3477	0.4223	0.3248	0.2989	0.3554	0.3078
	$\beta_{2}$	0.7807	0.8452	1.0728	0.8504	0.8087	0.9364	0.8155
	$\beta_{3}$	0.2224	0.2365	0.3044	0.2464	0.2173	0.2502	0.2322
	$\beta_{4}$	0.3997	0.4401	0.5791	0.4414	0.4106	0.4839	0.4214
300	$\beta_{0}$	0.1014	0.1042	0.1182	0.1008	0.0926	0.1079	0.0975
	$\beta_{1}$	0.1806	0.1930	0.2298	0.1829	0.1773	0.2112	0.1801
	$\beta_{2}$	0.5004	0.4792	0.5853	0.4814	0.4730	0.5560	0.4756
	$\beta_{3}$	0.1313	0.1315	0.1631	0.1383	0.1286	0.1501	0.1357
	$\beta_{4}$	0.2433	0.2449	0.3112	0.2485	0.2401	0.2856	0.2445

*Note:* Standard error estimated. The results are based on 500 Monte Carlo samples, considering the MC‐SD and the mean of the standard error estimates under methods AVE and SVE, for different numbers of subjects (n). The data were generated using an AR(2)‐ST‐LMM and fitted using the ST‐LMM, SN‐LMM, and T‐LMM models, all with AR(2) structure.

Abbreviations: AVE, asymptotic variance estimator; MC‐SD, Monte Carlo standard deviation; SVE, sandwich variance estimator.

The results indicate that the AVE method tends to overestimate standard errors when misspecification is present, compared to the MC‐SD. This tendency is more pronounced in smaller samples and fits performed with the SN‐LMM model, followed by T‐LMM. However, it is observed that this difference tends to decrease as the sample size increases. In contrast, the SVE method proved to be more robust to misspecification, generating standard error estimates closer to those of the MC‐SD, even in smaller samples. However, a small tendency to overestimate or underestimate is observed in some scenarios, especially when the SN‐LMM model is adjusted. These results indicate that, although robust, the method is not immune to the effects of misspecification of the random effects distribution.

## Appendix D Additional Results for Illustration Mice Weight Data

The 95% confidence intervals and their lengths for the fixed effects obtained using the AVE, bootstrap, and SVE methods considering different models are presented in Table A3. Regarding the interval lengths, the AVE method produced longer confidence intervals compared with the bootstrap and SVE methods.

**TABLE A3:** Mice Weight Data.

			AVE		Bootstrap		SVE		Interval length		
Structure	Parameter	Estimate	2.5%	97.5%	2.5%	97.5%	2.5%	97.5%	AVE	bootstrap	SVE
AR(1)‐N	$\beta_{0}$	39.471	38.185	40.756	38.367	40.235	38.414	40.527	2.571	1.868	2.113
	$\beta_{1}$	1.980	1.622	2.338	1.625	2.246	1.674	2.286	0.716	0.621	0.612
	$\beta_{2}$	−1.994	−3.810	−0.178	−3.061	−0.590	−3.436	−0.553	3.632	2.471	2.883
	$\beta_{3}$	−1.306	−3.733	1.120	−2.472	−0.047	−2.755	0.143	4.853	2.425	2.898
	$\beta_{4}$	−0.134	−0.674	0.406	−0.627	0.347	−0.629	0.362	1.080	0.974	0.991
	$\beta_{5}$	−0.702	−1.347	−0.057	−1.209	−0.244	−1.210	−0.194	1.290	0.965	1.017
AR(1)‐SN	$\beta_{0}$	39.255	37.949	40.561	38.310	39.975	38.341	40.169	2.612	1.665	1.828
	$\beta_{1}$	1.904	1.545	2.262	1.642	2.176	1.631	2.177	0.717	0.534	0.546
	$\beta_{2}$	−1.835	−3.655	−0.015	−3.105	−0.741	−3.207	−0.464	3.640	2.364	2.743
	$\beta_{3}$	−0.971	−3.405	1.463	−2.302	0.429	−2.342	0.399	4.868	2.731	2.740
	$\beta_{4}$	−0.059	−0.596	0.477	−0.526	0.324	−0.516	0.398	1.073	0.850	0.914
	$\beta_{5}$	−0.557	−1.269	0.155	−0.985	−0.099	−1.027	−0.086	1.424	0.886	0.941
AR(1)‐ST	$\beta_{0}$	39.537	38.131	40.944	38.687	40.476	38.745	40.329	2.813	1.789	1.583
	$\beta_{1}$	1.956	1.609	2.302	1.709	2.222	1.724	2.187	0.693	0.514	0.463
	$\beta_{2}$	−2.118	−4.071	−0.166	−3.168	−1.075	−3.230	−1.006	3.905	2.092	2.224
	$\beta_{3}$	−0.853	−2.624	0.917	−2.010	0.131	−1.986	0.279	3.541	2.140	2.266
	$\beta_{4}$	−0.149	−0.583	0.285	−0.485	0.222	−0.536	0.239	0.868	0.707	0.775
	$\beta_{5}$	−0.445	−0.991	0.100	−0.846	0.005	−0.834	−0.056	1.091	0.851	0.778
*AR(1)‐SSL*	$\beta_{0}$	39.863	38.438	41.288	38.625	41.077	39.092	40.633	2.850	2.452	1.541
	$\beta_{1}$	2.035	1.673	2.396	1.741	2.294	1.809	2.261	0.723	0.553	0.452
	$\beta_{2}$	−2.171	−4.218	−0.125	−3.194	−1.077	−3.257	−1.086	4.093	2.117	2.171
	$\beta_{3}$	−0.854	−2.623	0.916	−1.858	0.141	−1.962	0.255	3.539	1.998	2.217
	$\beta_{4}$	−0.169	−0.612	0.275	−0.551	0.206	−0.541	0.204	0.887	0.757	0.745
	$\beta_{5}$	−0.470	−1.015	0.075	−0.847	−0.127	−0.846	−0.094	1.090	0.721	0.752
UNC‐SSL	$\beta_{0}$	39.819	38.605	41.034	38.739	41.162	39.141	40.498	2.429	2.423	1.357
	$\beta_{1}$	2.011	1.704	2.319	1.759	2.314	1.803	2.220	0.615	0.555	0.416
	$\beta_{2}$	−1.637	−3.168	−0.106	−2.490	−0.721	−2.625	−0.649	3.062	1.769	1.976
	$\beta_{3}$	−0.280	−1.700	1.141	−1.263	0.655	−1.286	0.726	2.841	1.918	2.012
	$\beta_{4}$	−0.333	−0.663	−0.003	−0.622	−0.046	−0.639	−0.026	0.660	0.576	0.613
	$\beta_{5}$	−0.584	−0.961	−0.206	−0.860	−0.291	−0.888	−0.279	0.755	0.569	0.609
DEC‐SSL	$\beta_{0}$	39.917	38.426	41.408	38.678	41.196	39.138	40.696	2.982	2.518	1.558
	$\beta_{1}$	2.047	1.679	2.415	1.746	2.326	1.818	2.276	0.736	0.580	0.457
	$\beta_{2}$	−2.189	−4.244	−0.134	−3.190	−1.057	−3.284	−1.094	4.110	2.133	2.190
	$\beta_{3}$	−0.876	−2.647	0.896	−1.904	0.119	−1.984	0.232	3.543	2.022	2.216
	$\beta_{4}$	−0.171	−0.615	0.273	−0.556	0.209	−0.545	0.203	0.887	0.766	0.748
	$\beta_{5}$	−0.475	−1.056	0.105	−0.863	−0.165	−0.853	−0.097	1.161	0.697	0.756

*Note:* 95% confidence intervals and their lengths for the fixed effects model fit, obtained using the asymptotic variance estimator (AVE), bootstrap, and sandwich variance estimator (SVE) methods, considering different models.
